# Particle engineering enabled by polyphenol-mediated supramolecular networks

**DOI:** 10.1038/s41467-020-18589-0

**Published:** 2020-09-23

**Authors:** Jiajing Zhou, Zhixing Lin, Matthew Penna, Shuaijun Pan, Yi Ju, Shiyao Li, Yiyuan Han, Jingqu Chen, Gan Lin, Joseph J. Richardson, Irene Yarovsky, Frank Caruso

**Affiliations:** 1 ARC Centre of Excellence in Convergent Bio-Nano Science and Technology, and the Department of Chemical Engineering, The University of Melbourne, Parkville, VIC 3010 Australia; 2 School of Engineering, RMIT University, Melbourne, VIC 3001 Australia

**Keywords:** Chemistry, Materials science, Nanoparticles

## Abstract

We report a facile strategy for engineering diverse particles based on the supramolecular assembly of natural polyphenols and a self-polymerizable aromatic dithiol. In aqueous conditions, uniform and size-tunable supramolecular particles are assembled through π–π interactions as mediated by polyphenols. Owing to the high binding affinity of phenolic motifs present at the surface, these particles allow for the subsequent deposition of various materials (i.e., organic, inorganic, and hybrid components), producing a variety of monodisperse functional particles. Moreover, the solvent-dependent disassembly of the supramolecular networks enables their removal, generating a wide range of corresponding hollow structures including capsules and yolk–shell structures. The versatility of these supramolecular networks, combined with their negligible cytotoxicity provides a pathway for the rational design of a range of particle systems (including core–shell, hollow, and yolk–shell) with potential in biomedical and environmental applications.

## Abstract (web summary)

Monodisperse colloidal particles with tunable properties show promise for biomedical, energy, and environmental applications and simple routes for fabricating these particles are of interest. Here, the authors report a facile strategy for fabrication of diverse particles based on the supramolecular assembly of phenols and self-polymerizable thiols

## Introduction

Monodisperse colloidal particles with tunable shape, size, and composition show promise for biomedical, energy, and environmental applications^1–4^. The conventional toolkit for particle synthesis, including sol–gel, hydrothermal, microemulsion, and layer-by-layer methods, affords a myriad of functional nanoparticles and microparticles^5–8^. Still, simple synthetic routes for fabricating particles are of widespread interest because they afford engineered particle systems with distinct physical and chemical motifs^9,10^. Supramolecular complexation, which is widespread in nature and exploits the non-covalent dynamic interactions of simple precursors to assemble complex architectures, provides a promising pathway for the development of advanced materials with controlled structure and function^11–13^. However, this bottom-up approach for particle engineering requires a fine balance between the intrinsic supramolecular forces of assembly and the subsequent interfacial and stabilizing interactions, which has been an ongoing challenge^14–16^. To balance these supramolecular forces, synthetic approaches generally require the multi-step, rigorous synthesis of the supramolecular building blocks prior to assembly under exacting conditions^10,14,17,18^. Therefore, identifying readily available building blocks that are capable of assembling under ambient conditions with a high-degree of structural control and that exhibit an inherently high-binding affinity for a diverse range of materials will provide new opportunities for engineering complex nanostructured materials for various applications.

**Fig. 1:**
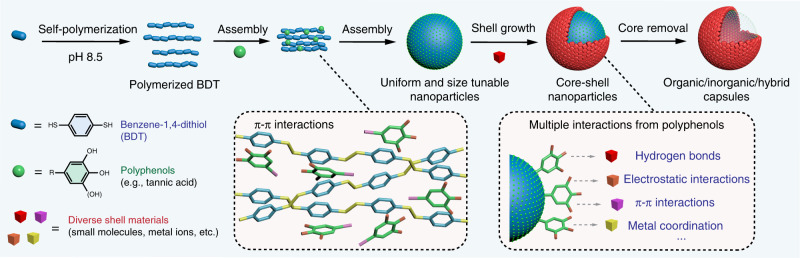
Synthetic strategy for particle engineering using supramolecular networks. Schematic of the synthesis of supramolecular particles through the controlled self-assembly of pBDT and polyphenols via π–π interactions. The polyphenol-stabilized particles allow for the subsequent growth of diverse shell materials on the particles. Disassembly of the supramolecular complexes in the core results in hollow structures.

In this work, a versatile supramolecular complexation strategy is presented to engineer physically and chemically diverse particles, as demonstrated by using natural polyphenols (e.g., tannic acid, TA) and a self-polymerizable aromatic dithiol (i.e., benzene-1,4-dithiol, BDT) (Fig. 1). Experimental work combined with molecular dynamics (MD) simulations show that in aqueous conditions, the presence of polyphenols facilitates the controlled self-assembly of polymerized BDT (pBDT) via π–π interactions, producing surface TA-stabilized pBDT supramolecular particles (pBDT–TA) ranging in size from ~50 to 650 nm. The pBDT–TA particles are colloidally stable in a range of aqueous environments (e.g., high salt, highly acidic, and alkaline environments) but can disassemble in organic solvents such as dimethylformamide (DMF). The presence of polyphenols on the particle surface facilitates the subsequent deposition of various materials (e.g., organic, inorganic, and hybrid) by exploiting the adherent nature of phenolic moieties, enabling the engineering of a variety of monodisperse particles with distinct structures (e.g., core–shell, hollow, and yolk–shell). This generalizable approach provides a platform for synthesizing diverse particles with structural and compositional complexities.

## Results

### Synthesis of pBDT–TA supramolecular particles

**Fig. 2:**
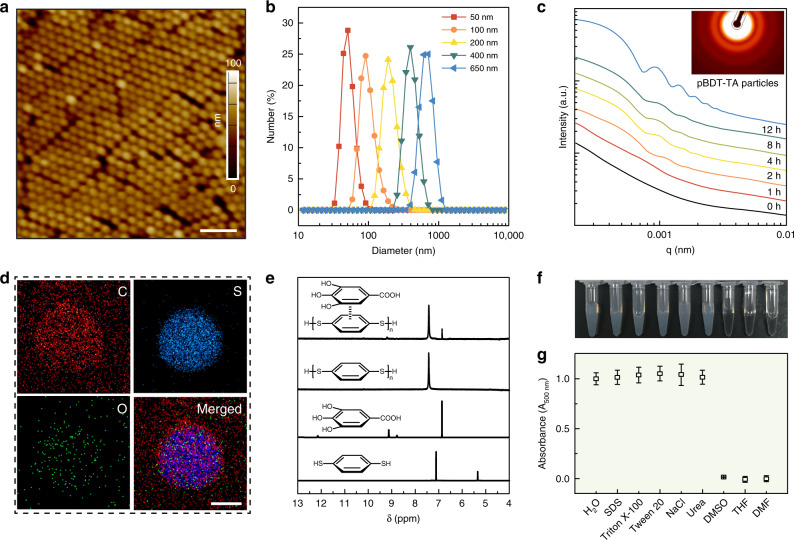
Assembly and disassembly of supramolecular pBDT–polyphenol particles. **a** AFM image of 100 nm pBDT–TA particles prepared from a mixture of 0.5 mg mL^−1^ BDT and 0.5 mg mL^−1^ TA. Scale bar is 500 nm. **b** Dynamic light-scattering data of the pBDT–TA particles with different sizes. **c** In situ growth of the pBDT–TA particles, as measured by SAXS. Inset: SAXS pattern of the pBDT–TA particles obtained after 12 h of reaction, post-purification. **d** EDX elemental mapping of the pBDT–TA particles. Scale bar is 50 nm. **e** ^1^H NMR spectra of the different components in the complexes. **f**, **g** Photograph and turbidity results of the pBDT–TA particles (100 nm) in different media. The error bars represent standard deviations (*n* = 3).

The primary advantage of the reported assembly method is that uniform, adherent supramolecular particles can be synthesized simply by mixing BDT and TA in bicine buffer (pH 8.5) at ambient temperature. Increasing the BDT-to-TA ratio led to larger pBDT–TA particles, as observed by atomic force microscopy (AFM) and transmission electron microscopy (TEM) (Fig. 2a and Supplementary Fig. 1). Specifically, the size of the particles increased from 50 to 150 nm by increasing the concentration of BDT from 0.25 to 1.0 mg mL^−1^ while fixing the concentration of TA at 0.5 mg mL^−1^ (Fig. 2b). Time-dependent dynamic light-scattering results showed that the particles started to form within 10 min after mixing BDT and TA (Supplementary Fig. 2), after which time they gradually continued to grow into larger pBDT–TA particles over the next 12 h. Supramolecular particles with larger diameters (>150 nm) were prepared by simply repeating the assembly process around the pBDT–TA seeds (Fig. 2b and Supplementary Fig. 3). In situ small-angle X-ray scattering (SAXS) measurements revealed the kinetics of the assembly process, wherein the minimum *q* shifted to lower values as the particles grew larger in size. The form factor oscillation of the pBDT–TA particles displayed at least five minima (Fig. 2c), which along with dynamic light scattering data confirmed a relatively narrow size distribution (polydispersity index (PDI) < 0.05)^19^.

Energy-dispersive X-ray spectroscopy (EDX) mapping and X-ray photoelectron spectroscopy results revealed that the pBDT–TA particles were composed of C, S, and O (Fig. 2d and Supplementary Fig. 4). The TA composition in the supramolecular networks was further calculated as ~9.2 wt% based on the mass of O. Following self-polymerization, Fourier transform infrared spectroscopy showed the disappearance of the υ_S–H_ stretching mode at 2559 cm^−1^ (Supplementary Fig. 5), indicating linkage of the thiol groups and polymerization of BDT^20^. This was also confirmed by the negligible residual BDT monomers in the pBDT–TA particles as well as their significantly improved thermal stability relative to that of TA or BDT (Supplementary Figs. 6 and 7). The importance of the disulfide linkages in the assembly process was examined using monothiol-carrying benzene molecules (e.g., thiophenol)^21^; however, only irregular aggregates were produced, as deduced from the large PDI values (Supplementary Fig. 8). Notably, our current aqueous-based polymerization of thiols using air as the oxidant at ambient temperature is an environmentally friendly assembly route compared to typical thiol polymerization reactions that proceed under stringent conditions (Supplementary Fig. 9)^22^. Notably, the hydrophobic pBDT aggregated by itself after reaching a certain chain length and precipitated from the solution if polyphenols are absent (Supplementary Fig. 9).

### Supramolecular interactions in the complexes

Various polyphenol species (i.e., gallic acid (GA), catechin (CAT), and epigallocatechin gallate (EGCG)) were studied as alternatives to TA for preparing supramolecular particles and elucidating the fundamental supramolecular interactions owing to their molecularly defined nature (Supplementary Figs. 10 and 11). The absence of new peaks or chemical shift from nuclear magnetic resonance (NMR) spectroscopy results revealed that the polyphenol (i.e., GA) did not undergo any chemical changes after forming the assemblies (Fig. 2e), indicating that the pBDT was assembled with the phenolics via noncovalent interactions rather than covalent bonds. Furthermore, all of the obtained particles had a surface zeta potential ranging from −30 to −40 mV in water (Supplementary Table 1), suggesting that the surface was composed primarily of polyphenols^16,23^. Such pBDT–TA particles were stable in solutions of 100 mM sodium dodecyl sulfate (SDS), Tween 20, Triton X-100, NaCl, or urea, but could readily disassemble in organic solvents such as dimethyl sulfoxide (DMSO), tetrahydrofuran (THF), and DMF (Fig. 2f, g and Supplementary Fig. 12). Moreover, the pBDT–TA particles remained stable in harsh environments, such as high pH (pH 13), low pH (pH 1), and high temperature (i.e., 100 °C) (Supplementary Fig. 13), suggesting that π–π interactions are responsible for stabilizing the assemblies.

Compared to other polyphenol-based supramolecular systems, such as chelation-based nanoparticles (NPs)^24^, the pBDT–TA supramolecular networks allow tailoring of particle size, and they are stable in harsh aqueous environments (chelation-based materials typically disassemble in acid conditions^25^). Thus, the pBDT–TA particles are envisioned to provide additional versatility and functionality for engineering NPs, while also affording a number of opportunities for subsequent modification.

### MD simulation of the assembly and disassembly of complexes

**Fig. 3:**
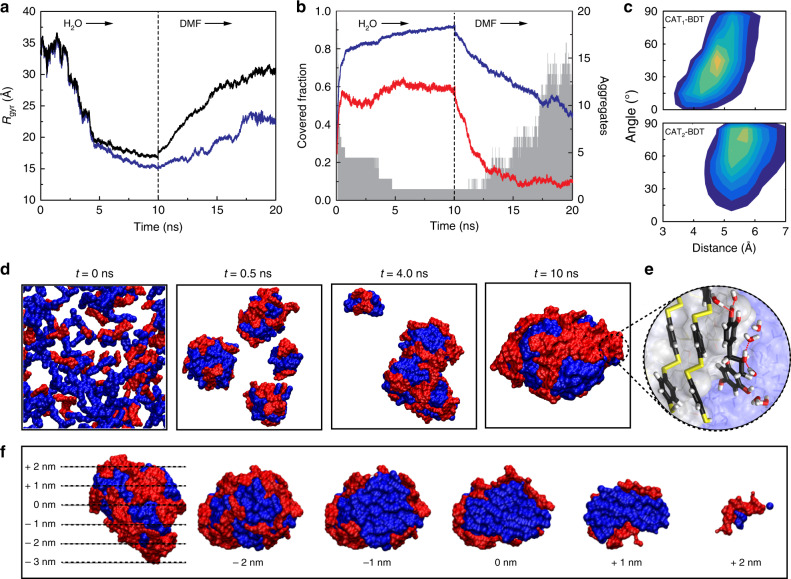
All-atom MD simulation of the assembly and disassembly of pBDT and CAT in water and DMF, respectively. **a** Radius of gyration (*R*_gyr_) of pBDT (blue) and pBDT–CAT (black). **b** Covered fraction (namely the fraction of molecules not accessible to the solvent) of pBDT (blue) and CAT (red) molecules, and number of pBDT aggregates (gray). **c** Population distribution of the angle and distance between the plane of the aromatic group of each CAT molecule and a BDT aromatic ring. **d** Evolution profiles of the size of the complex, comprised of pBDT (blue) and CAT (red), showing aggregates at 0, 0.5, 4.0, and 10 ns of MD simulation in water. MD snapshot of the largest formed single aggregate at 10 ns. **e** Magnified interface section showing molecular details of aromatic stacking of BDT and CAT and hydrogen bonding between CAT and water; carbon, oxygen, hydrogen, and sulfur are colored black, red, white, and yellow, respectively, and hydrogen bonds are represented by thin red lines. **f** Cross-sectional images of the pBDT–CAT complex after 10 ns of MD simulation in water highlighting the pBDT core (blue) and CAT shell (red). 0 nm indicates the center of mass of the complex in the vertical direction (left snapshot) and cross-sections rotated 90° are shown within the −2 to 2 nm range.

All-atom MD simulations were used to study the growth of the pBDT–polyphenol complexes over time and to quantify the evolution of the radius of gyration (*R*_gyr_) and the solvent-accessible surface area (SASA) of the polyphenols and pBDT complex systems (Fig. 3a, b, Supplementary Figs. 14 and 15, and Supplementary Table 2). For these simulations, GA and CAT were selected as representative polyphenols owing to the simplicity of the molecules (one and two aromatic rings, respectively) and their use in our empirical experiments. In all-atom MD simulations of the pBDT–CAT system, the SASA of CAT stabilized at ~50% (of the total CAT SASA), whereas the SASA of the pBDT portion approached ~100% as the complexes stabilized within the timeframe of the simulation. MD simulations demonstrated that pBDT mainly forms the core of the complexes with the polyphenols comprising an outer layer of the particles (Fig. 3d and Supplementary Movie 1). Cross-sectional slices taken at different distances from the center of mass of the complex emphasize the hydrophobic core–polyphenol shell architecture (Fig. 3f). These results suggest that co-precipitation is a key driving force behind assembly. The simulations further highlighted that the phenolic molecules can interact with BDT through π–π stacking without compromising their ability to form hydrogen bonds with the (aqueous or other hydrophilic) environment and among themselves (Fig. 3c, e, Supplementary Fig. 16, and Supplementary Table 3). This explains the experimentally observed high colloidal stability of the particles with no appreciable aggregation over 6 months at room temperature (25 °C) (Supplementary Fig. 17)—the polyphenols can provide bridging interactions between pBDT and water. In addition, the MD simulation showed that the π–π interactions in the simulated complexes are easily destabilized in organic solvents (Fig. 3a, b, and Supplementary Movie 2), as observed experimentally. Similar assembly and disassembly behaviors were observed in the GA-based system (Supplementary Fig. 18 and Supplementary Table 4), indicating the generic formation mechanism for such supramolecular networks.

### Secondary coating for engineering diverse particles

**Fig. 4:**
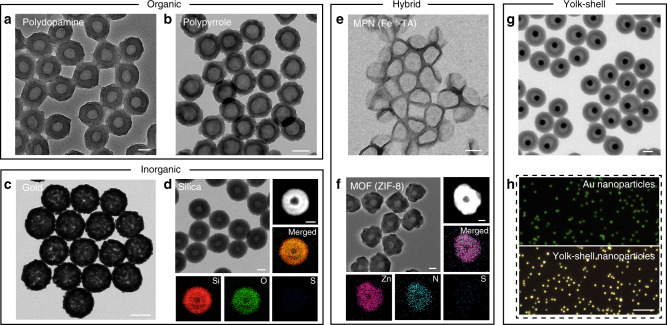
Synthesis of hollow particles using sacrificial pBDT–TA particles. **a**–**f** TEM images of hollow structures after removal of the pBDT–TA core. Insets in **d** and **f** are HAADF-STEM images of representative hollow particles and their corresponding EDX mapping results. **g** TEM image of AuNP@polydopamine yolk–shell NPs. **h** Dark-field images of 50 nm AuNPs and AuNP@polydopamine yolk–shell NPs. Scale bars are 100 nm (**a**–**g** main images and insets) and 10 µm **h**.

Phenolic groups, such as gallol and catechol moieties, are known to exhibit a strong affinity with a broad range of materials via noncovalent interactions (e.g., hydrogen bonding, π–π interactions, coordination bonds, electrostatics)^26,27^. The MD simulation results, showing the localization of the phenolic molecules at the interface of the particle and water, suggest that the phenolic molecules on the surface could enable further interactions with building blocks to generate compositionally complex core–shell particles. To validate this hypothesis, various precursors (such as organic molecules, silica precursors, and metal salts) were deposited on the pBDT–TA particles under distinct synthetic conditions. For example, organic (e.g., polydopamine, polypyrrole), inorganic (e.g., silica, Au), and hybrid organic/inorganic (e.g., metal–phenolic network (MPN), metal–organic framework (MOF)) materials were successfully coated on the pBDT–TA particles (Supplementary Fig. 19), yielding a library of monodisperse core–shell particles. Additionally, the pBDT–TA particles can serve as sacrificial templates to generate hollow particles when the coated particles are immersed in DMF (Fig. 4). The low S signal in EDX indicated that the complexes were successfully removed during the disassembly process, and the defined voids in the high-angle annular dark-field-scanning transmission electron microscopy (HAADF-STEM) images matched the size of the original pBDT–TA particles (Fig. 4d, f, and Supplementary Fig. 20). Importantly, the resultant shells retained the inherent properties of the coating material (e.g., crystal structure), suggesting that the mild removal process did not significantly damage the coating material (Supplementary Fig. 21). Hollow particles with different structural parameters (e.g., shell thickness (15–25 nm) and inner diameter (100–200 nm)) were also prepared using the present pBDT–TA-mediated strategy (Supplementary Figs. 22 and 23).

These supramolecular networks can be applied as colorless coatings to modify a wide variety of macroscopic substrates (such as polydimethylsiloxane, glass, Au, and stainless steel mesh) (Supplementary Figs. 24–27). Furthermore, they can be applied to nanoscale substrates (such as AuNPs and iron oxide (IO) NPs) (Supplementary Fig. 28). Owing to the characteristic localized surface plasmon resonance (LSPR) feature of AuNPs, the successful coating on AuNPs was also validated by UV–vis spectra—a red shift of the LSPR from 535 nm (AuNPs) to more than 600 nm (Supplementary Fig. 29) was observed. The combination of the graft to and graft from properties of BDT–TA complexes can afford the possibility of generating yolk–shell structures. As a proof of concept, by using pBDT–TA as both a coating (on AuNPs) and a removable template (for polydopamine), yolk–shell structures with structural complexity were readily engineered (Fig. 4g and Supplementary Fig. 30). The obtained yolk–shell particles were therefore used as dark-field imaging probes due to the enhanced scattering signals (Fig. 4h)^28^. Notably, the pBDT–TA particles showed negligible cytotoxicity (Supplementary Fig. 31, Supplementary Methods), suggesting their potential for biomedical and environmental applications^29,30^.

We have developed a versatile supramolecular complex-enabled strategy for the synthesis of a variety of particles. Central to this general and robust pathway is the facile assembly and disassembly of pBDT–TA complexes. The adhesive nature of polyphenols allows for the growth of various coating materials on pBDT–TA. Importantly, the pBDT–TA complexes showed negligible cytotoxicity. We anticipate that this interface engineering platform will underpin the development of a wide range of intricate nanostructures including core–shell, hollow, yolk–shell, and onion-like particles, where the resultant particles can serve as model systems for fundamental research in nanoscience and open up new avenues in diverse applications.

## Methods

### Synthesis of pBDT–TA particles

For the synthesis of pBDT–TA particles of 100 nm, 1.5 mL TA solution (4 mg mL^−1^ in water) was first added to 12 mL bicine buffer (pH 8.5, 10 mM). After vigorous stirring for 10 min, 1.5 mL BDT solution (4 mg mL^−1^ in DMSO) was added to the above solution. The reaction was stirred for 12 h. Then, the resulting particles were purified by centrifugation (10,000 × *g*, 10 min) twice to remove the excess complexes. The pellet was resuspended in water for future use. The size of the particles could be tuned from 50 to 150 nm by varying the concentration of BDT from 0.25 to 1.0 mg mL^−1^ while fixing the concentration of TA at 0.5 mg mL^−1^.

### Seed-mediated growth of pBDT–TA particles of large diameters

For the synthesis of large pBDT–TA particles (e.g., 200 nm), 100 µL of the smaller pBDT–TA particles (100 nm) was added to 12 mL bicine buffer (pH 8.5, 10 mM) under stirring. Then, 1.5 mL TA solution (4 mg mL^−1^ in water) was added, followed by dropwise addition of 1.5 mL BDT solution (4 mg mL^−1^ in DMSO). After reaction for 12 h, the resulting large pBDT–TA particles were purified by centrifugation (8000 × *g*, 10 min) twice to remove excess complexes. The pellet was resuspended in water for future use. For the synthesis of larger pBDT–TA particles (>200 nm), multiple assembly steps were performed to achieve the required particle size.

### Synthesis of particles using different polyphenols

Typically, 1.5 mL polyphenol solution (4 mg mL^−1^ in water) was first added to 12 mL bicine buffer (pH 8.5, 10 mM). After vigorous stirring for 10 min, 1.5 mL BDT solution (4 mg mL^−1^ in DMSO) was added to the above solution. The solution was stirred for 12 h. Four different types of polyphenols, namely, TA, GA, CAT, and EGCG, were used to prepare the supramolecular particles.

### Synthesis of particles using different thiol-carrying benzene molecules

Typically, 1.5 mL TA solution (4 mg mL^−1^ in water) was first added to 12 mL bicine buffer (pH 8.5, 10 mM). After vigorous stirring for 10 min, a solution of thiol-carrying benzene molecules (4 mg mL^−1^ in DMSO) was added to the above solution. The solution was stirred for 12 h. In this experiment, four different types of thiol-carrying benzene molecules, namely, BDT, thiophenol, 4-mercaptobenzoic acid, and 4-aminothiophenol, were used to prepare the complexes.

### Stability of pBDT–TA particles in different solvents

Typically, pBDT–TA particles were dissolved in 1.6 mL 100 mM SDS, 100 mM Triton X-100, 100 mM Tween-20, 100 mM NaCl, 100 mM urea, DMSO, THF, or DMF. The solution was vigorously mixed by a vortex mixer for 5 s and then incubated for 1 h. The turbidity was recorded by an Analytik Jena SPECORD 250 instrument. The extinction spectra were collected from 400 to 600 nm. To assess the stability of the particles in solutions of Dulbecco’s phosphate-buffered saline, 100 mM NaOH, 100 mM HCl, and under elevated temperatures (100 °C), the particles were purified by centrifugation (10,000 × *g*, 10 min) after 1 h of incubation. Then, the size of the particles was measured using a Malvern NANO-ZS90 Zetasizer.

### Synthesis of polydopamine-coated pBDT–TA particles

Typically, 500 µL of the concentrated pBDT–TA particles was dispersed in 10 mL bicine buffer (10 mM, pH 8.5), followed by the addition of 1.5 mL dopamine solution (4 mg mL^−1^ in water). The reaction solution was stirred for 12 h and the resulting brown product was purified by centrifugation (10,000 × *g*, 10 min) twice. The pellet was redispersed in water for future use.

### Synthesis of polypyrrole-coated pBDT–TA particles

Typically, 20 µL of pBDT–TA particles was washed with water and redispersed in 600 µL water. Then, 200 µL 0.5% SDS was added to the mixture. After brief vortexing, 200 µL pyrrole (4 mg mL^−1^ in water) and 100 µL ammonium persulfate were added to induce polymerization of pyrrole on the surface of the pBDT–TA particles. The mixture was shaken at room temperature for 12 h. The green product was purified by centrifugation (9000 × *g*, 10 min) twice. The pellet was redispersed in water for future use.

### Synthesis of silica-coated pBDT–TA particles

Typically, 2.5 mL cetyltrimethylammonium bromide (15 mM) was dissolved in 10 mL H_2_O. After stirring for 10 min, 3 mL isopropanol was added, followed by the addition of 500 µL pBDT–TA particles in water. The mixture was stirred for 10 min. Then, 300 µL ammonia solution (25%) was added, followed by the addition of 200 µL tetraethyl orthosilicate (20% in methanol (MeOH)). The reaction was stirred for 12 h. The silica-coated pBDT–TA particles were collected by centrifugation (7000 × *g*, 10 min). The particles were washed with MeOH twice by centrifugation/redispersion. The pellet was redispersed in water for future use.

### Synthesis of Au-coated pBDT–TA particles

Au seeds of around 3.5 nm in diameter were first synthesized for facilitating the growth of a Au shell. In brief, 600 µL HAuCl_4_·3H_2_O (25 mM) and 600 µL sodium citrate (25 mM) were sequentially injected into 60 mL water. After 10 min of stirring, 900 µL freshly prepared NaBH_4_ (0.2 M) was injected into the mixture. The color changed immediately from pale yellow to reddish orange, indicating the successful synthesis of Au seed colloids. The resultant solution was stirred for 15 min and aged in the dark for at least 2 h prior to subsequent experiments. Note that only freshly prepared Au seed colloids should be used for obtaining the Au nanoshells.

pBDT–TA particles were then surface-modified with the Au seeds prior to the growth of the shell. In a typical synthesis, pBDT–TA particles were dispersed in water and sonicated for at least 5 min. Under continuous sonication, a freshly prepared Au seed solution was injected into the particle suspension. Then, the seeded pBDT–TA particles were purified by centrifugation (9000 × *g*, 10 min) four times to remove excess Au seed colloids. The obtained seeded pBDT–TA particles were redispersed in water and were stored at 4 °C for future use.

The seeded pBDT–TA particles were used to synthesize the Au shell. Typically, 50 μL pBDT–TA particles with seeds on the surface was added to 2 mL of H_2_O. After stirring for 2 min, 100 μL 2.5 mM KAuCl_4_ was injected, followed by the addition of 100 μL of 0.2 M NH_2_OH·HCl. The color of the solution changed from light red to brownish green immediately. The reaction solution was stirred for 2 min. After cooling, 100 μL mercaptopolyethylene glycol monomethyl ether (5 kDa, 10 mg mL^−1^) was added to the solution to further stabilize the Au shell. Finally, the product was purified by centrifugation (700 × *g*, 10 min).

### Synthesis of MPN-coated pBDT–TA particles

The assembly of an MPN (i.e., Fe^III^–TA) coating on the particle template was based on the protocol therein with some minor modifications^31^ detailed as follows. Briefly, 100 μL pBDT–TA particles was first dispersed in 300 µL water. Then, 10 μL TA solution (24 mM) and 10 µL FeCl_3_·6H_2_O (37 mM) were successively added to the particle suspension at room temperature followed by brief vortexing and sonication after each addition. The pH was raised by promptly adding 500 μL 3-(*N*-morpholino)propanesulfonic acid buffer (20 mM, pH 7.4), and the solution was thoroughly vortexed for 5 min to allow sufficient film formation and adherence. The obtained particles coated by the MPN were washed three times with water to remove excess materials by centrifugation (9000 × *g*, 10 min). During each washing step, the supernatant was removed, and the pellet was vortexed and sonicated for 30 s to avoid particle aggregation. The pellet was resuspended in water for future experiments.

### Synthesis of MOF-coated pBDT–TA particles

Typically, 200 µL pBDT–TA particles was dispersed in MeOH and centrifuged once to remove residual water in the suspension. Then, 1 mL 2-methylimidazole (25 mM in MeOH) was added. The solution was vigorously mixed by a vortex mixer for 10 s, followed by the addition of 1 mL of Zn(NO_3_)_2_ (12.5 mM in MeOH). The mixture was shaken for 10 s and incubated at room temperature for 12 h. The pBDT–TA particles coated by an MOF material, i.e., zeolitic imidazolate framework-8 (ZIF-8), were collected by centrifugation (8000 × *g*, 10 min) and washed three times with MeOH before drying under vacuum overnight.

### Disassembly of pBDT–TA particles to produce hollow particles

Typically, 50 µL of the pBDT–TA particles with a shell coating was incubated with 1.6 mL DMF at 37 °C. The dispersion was shaken at 600 rpm for 12 h to complete the disassembly of the pBDT–TA particles. The hollow particles were then collected by centrifugation. Different hollow particles required different conditions (i.e., hollow polydopamine (18,000 × *g*, 10 min), hollow polypyrrole (18,000 × *g*, 10 min), hollow silica (3000 × *g*, 10 min), hollow Au (800 × *g*, 10 min), hollow MPN (13,000 × *g*, 10 min), and hollow ZIF-8 (3000 × *g*, 10 min)).

### Fabrication of pBDT–TA complex coatings on diverse substrates

Prior to use, the poly(dimethylsiloxane), glass, Au, and stainless steel wire mesh substrates were rinsed with water three times to obtain a clean surface. Typically, the substrate was immersed in 12 mL bicine buffer (pH 8.5, 10 mM), followed by the sequential addition of 1.5 mL TA solution (4 mg mL^−1^ in water), and 1.5 mL BDT solution (4 mg mL^−1^ in DMSO). After vigorous stirring for 10 min, the stirring rate was reduced to 50 rpm. The reaction was stirred for 18 h. Then, the substrate was washed with water three times. The substrates were coated with pBDT–TA complexes twice to obtain a thicker coating so that they could be easily observed by the naked eye.

### Synthesis of AuNP@pBDT–TA core–shell particles (50 nm AuNPs)

AuNPs of 50 nm in diameter were prepared using a seeded growth method^32^ as follows. AuNPs (seeds) with a diameter of 20 nm were first synthesized: 5 mL sodium citrate solution (12 mg mL^−1^) was injected into 50 mL boiling water solution containing 5 mg HAuCl_4_ under vigorous stirring. A color change from colorless to red was observed within 5 min. The solution was heated for another 30 min resulting in Au seeds with a diameter of 20 nm, which were then cooled to room temperature before further use. Then, the AuNPs of 20 nm in diameter were used to synthesize AuNPs of 50 nm in diameter. Briefly, 50 mL water was added into a 100 mL round-bottom flask. Then, 2 mL of the seed solution (20 nm AuNPs) and 200 μL of 0.2 M NH_2_OH·HCl were consecutively added to this flask. Subsequently, 3 mL of 0.1 wt% HAuCl_4_ was added dropwise to the solution under vigorous stirring followed by reaction for 30 min at room temperature. A gradual color change from light red to dark red was observed. Finally, the concentration of sodium citrate was adjusted to 1 mM. After reacting for another 2 h, the nanoparticle dispersion was stored at 4 °C for further use.

For the synthesis of AuNP@pBDT–TA with a 50 nm Au core, AuNPs (optical density: 35) were first added to 12 mL bicine buffer (pH 8.5, 10 mM). After vigorous stirring for 10 min, 1.5 mL TA solution (4 mg mL^−1^ in water) was added. Then, 1.5 mL BDT solution (4 mg mL^−1^ in DMSO) was added dropwise to the above solution. The reaction was stirred for 12 h. The solution was purified by centrifugation (1200 × *g*, 10 min) to remove excess complexes. For the washing process, the NPs were spun down by centrifugation and the supernatant was removed. The pellet was resuspended in water for future use. The thickness of the complexes can be tuned from 5 to 40 nm by varying the concentration of BDT from 1 to 8 mg mL^−1^.

### Synthesis of AuNP@pBDT–TA core–shell particles (14 nm AuNPs)

AuNPs of 14 nm in diameter were prepared by citrate reduction of HAuCl_4_ in aqueous phase^33^ as follows. Typically, sodium citrate (102 mg) in water solution (2 mL) was rapidly injected into a boiling aqueous HAuCl_4_ solution (30 mg in 200 mL water) under vigorous stirring. After boiling for 15 min, the solution was cooled to room temperature.

For the synthesis of AuNP@pBDT–TA with a 14 nm Au core, AuNPs (optical density: 20) were first added to 12 mL bicine buffer (pH 8.5, 10 mM). After vigorous stirring for 10 min, 1.5 mL TA solution (4 mg mL^−1^ in water) was added. Then, 1.5 mL BDT solution (4 mg mL^−1^ in DMSO) was added dropwise to the above solution. The reaction was stirred for 12 h. The solution was purified by centrifugation (10,000 × *g*, 10 min). For the washing process, the NPs were spun down by centrifugation and the supernatant was removed. The pellet was resuspended in water for future use.

### Synthesis of IO@pBDT–TA core–shell particles

For the synthesis of IO@pBDT–TA with an IO core, IO NPs (2 mg mL^−1^) were first added to 12 mL bicine buffer (pH 8.5, 10 mM). After vigorous stirring for 10 min, 1.5 mL TA solution (4 mg mL^−1^ in water) was added. Then, 1.5 mL BDT solution (4 mg mL^−1^ in DMSO) was added dropwise to the above solution. The reaction was stirred for 12 h. The solution was purified by centrifugation (16,000 × *g*, 10 min). For the washing process, the NPs were spun down by centrifugation and the supernatant was removed. The pellet was resuspended in water for future use.

### Synthesis of AuNP@polydopamine yolk–shell particles

Typically, AuNP@pBDT–TA NPs were first dispersed in 12 mL bicine buffer (pH 8.5, 10 mM) under vigorous stirring. Then, 1.5 mL dopamine solution (4 mg mL^−1^ in water) was added dropwise. The reaction solution was stirred for 12 h and the product was purified by centrifugation (1200 × *g*, 10 min). For the washing process, the NPs were spun down by centrifugation and the supernatant was removed. The pellet was redispersed in water for future use. The thickness of the shell could be modulated from 5 to 50 nm by varying the volume of the dopamine solution from 0.5 to 3.5 mL.

To obtain the yolk–shell particles, the pBDT–TA layer was dissolved by DMF. In brief, 50 µL of the AuNP@pBDT–TA@polydopamine particles was incubated in 1.6 mL DMF at 37 °C. The solution then was shaken at 600 rpm for 12 h to allow the pBDT–TA complexes to disassemble completely. The hollow particles were then collected by centrifugation (2000 × *g*, 10 min).

### Model construction

Initial conformations of the polyphenol and BDT mixtures were randomly packed into a 3D simulation cell^34^ with composition and densities indicated in Supplementary Table 2. Two polyphenols, i.e., GA and CAT, were chosen as representative models of typical chemical structures of polyphenols (e.g., catechol and pyrogallol groups); furthermore, CAT maintains a certain level of molecular flexibility compared to TA. Monomers (BDT_1_), oligomers with three repeating units (BDT_3_) and six repeating units (BDT_6_) were chosen to study the role of BDT polymerization in the assembly process.

The generated mixed systems were immersed in a pre-equilibrated box of water molecules. Water molecules within 2.5 Å of either polyphenol or BDT_1_/BDT_3_/BDT_6_ molecules were removed and spontaneous MD simulations performed to model the self-assembly process as described below. Initial structures for the disassembly simulations were taken from the final 0.5 ns of the assembly simulations and the BDT_*n*_/polyphenol aggregates were inserted into a pre-equilibrated box of either DMSO or DMF. Solvent molecules within 2.5 Å of either polyphenol or BDT_*n*_ molecules were removed. Simulations of a single GA or CAT molecule were run for 1.1 ns to generate comparison statistics for hydrogen-bonding analysis. All systems simulated are shown in Supplementary Table 2.

### Simulations

All simulations were carried out using Forcite MD code from Biovia, Dassault Systèmes^35^. The COMPASS II force field was used for all simulations^36^. Nonbonded interactions were calculated using the atom-based summation method with a cutoff radius of 12.5 Å, a spline width of 2.5 Å, and a buffer width of 1.0 Å. A long-range vdW tail correction was applied for nonbonded interactions longer than the cutoff radius. Long-range electrostatic interactions were calculated using the particle-mesh Ewald method. The conjugate gradient algorithm was used for energy minimization, with an energy convergence criterion of 0.01 kcal mol^−1^ Å^−1^. For MD procedures, a 1.0 fs time step was used for the simulation of the NPT ensemble, using the Nose thermostat to control the temperature at 298 K and the Berendsen barostat to control pressure at 1 atm^37,38^. System coordinates were output every 1 ps. Assembly simulations were performed for 2.5–12 ns until stable aggregates were observed. Cross-sectional slices were taken in 1 nm increments from the center of mass of the complex after 10 ns. Disassembly simulations were performed between 3 and up to ~10 ns until the disassembly became evident. Statistical analysis was performed over the final 0.5 ns of the self-assembly trajectories to produce property distributions and averages.

### Analysis

Aggregate data were determined using a distance criterion between non-hydrogen atoms in BDT molecules. A BDT molecule is defined to be in an aggregate if a non-hydrogen atom of the molecule is within 0.4 nm of another BDT molecule in the aggregate.

Angle against distance population distributions were generated over the final 0.5 ns for each of the assembly simulations. The angle between the two aromatic rings was calculated as the angle between the two planes that the aromatic rings lie in. The atoms used to define the planes are shown in Supplementary Fig. 16. The distance between two aromatic rings was calculated as the distance between the centers of mass of the atoms used to define the plane of the aromatic rings. Owing to the two aromatic rings present in CAT, distributions are separated into CAT_1_ → BDT, where CAT_1_ is the aromatic ring in each CAT closest to a BDT ring (for each CAT molecule this can either be CATR1 or CATR2), and CAT_2_ → BDT, where CAT_2_ is the other aromatic ring for each catechin molecule.

## Supplementary information

Supplementary Information

Description of Additional Supplementary Files

Supplementary Movie 1

Supplementary Movie 2

## Data Availability

The authors declare that the data supporting the findings in this study are available in the paper and its supplementary information files. Additional data related to this paper that further support the findings of this study are available from the corresponding author upon reasonable request.
