# Supplementary material for: Particle engineering enabled by polyphenol-mediated supramolecular networks
Source: Nat Commun. 2020 Sep 23;11:4804. doi: 10.1038/s41467-020-18589-0 (PMC7511334; doi:10.1038/s41467-020-18589-0)
Supplement: Supplementary file 1 — Supplementary Information [file 41467_2020_18589_MOESM1_ESM.pdf]

Supplementary Information for

Particle engineering enabled by polyphenol-mediated supramolecular networks

Zhou et al.

Supplementary Methods

Materials and Methods

Materials

Tannic acid (TA, ACS reagent), bicine (≥99%), 3-(*N*-morpholino)propanesulfonic acid (MOPS, ≥99.5%), gallic acid (GA, 97.5−102.5%), epigallocatechin gallate (EGCG, ≥95%), catechin (CAT, ≥99%), benzene-1,4-dithiol (BDT, 99%), thiophenol (97%), 4-mercaptobenzoic acid (99%), 4-aminothiophenol (97%), sodium dodecyl sulfate (SDS, ≥99%), Triton X-100 (BioXtra), Tween-20 (BioXtra, viscous liquid), urea (99.0–100.5%), sodium chloride (NaCl, ≥99.5%), dopamine, pyrrole (98%), ammonium persulfate (APS, ≥98.0%), cetyltrimethylammonium bromide (CTAB, ≥98.0%), ammonia solution (25%), tetraethyl orthosilicate (TEOS, 98%), sodium borohydride ($NaBH_4$, ≥98.0%), gold(III) chloride trihydrate ($HAuCl_4 \cdot 3H_2O$, ≥99.9%), potassium gold(III) chloride ($KAuCl_4$, 99.99%), hydroxylamine hydrochloride ($NH_2OH \cdot HCl$, 98%), iron(III) chloride hexahydrate ($FeCl_3 \cdot 6H_2O$, ≥98.0%), zinc nitrate hexahydrate ($Zn(NO_3)_2 \cdot 6H_2O$, 98%), 2-methylimidazole (99%), hydrogen peroxide solution (H_2O_2, 30 wt.%), sulfuric acid (H_2SO_4, 98%), hydrochloric acid (HCl, 36.5–38.0%), sodium hydroxide (NaOH, ≥98%), Sylgard184, sodium citrate (≥99%), Dulbecco's phosphate-buffered saline (DPBS), and phenazine methosulfate (PMS) were purchased from Sigma-Aldrich. Mercaptopolyethylene glycol monomethyl ether (mPEG-SH, 5 kDa) were purchased from JenKem Technology USA Inc. Iron oxide (IO) nanoparticles modified with 25 kDa mPEG (50 nm in diameter) were purchased from micromod Partikeltechnologie GmbH. Methanol (MeOH), ethanol (EtOH), dimethyl sulfoxide (DMSO), tetrahydrofuran (THF), dimethylformamide (DMF), and isopropanol (IPA) were purchased from Chem Supply. Planar glass substrates (76 mm × 26 mm) were obtained from Waldemar Knittel. Poly(dimethylsiloxane) (PDMS) substrates were prepared by mixing Sylgard184 base and curing agent (10:1 w/w ratio, Dow Corning) followed by curing at room temperature for 24 h. Planar quartz substrates (76 mm × 25 mm) were purchased from ProSciTech. Gold surfaces were prepared using an Emitech K575x sputter coater. Stainless steel meshes (400 × 400 openings per square inch) were purchased from McMaster-Carr. High-purity water with a resistivity of 18.2 MΩ cm was obtained from an inline Millipore RiOs/Origin water purification system. All solutions were freshly prepared for immediate use in each experiment. Raw cells and THP-1 cells were purchased from the American Type Culture Collection (USA). Dulbecco's modified Eagle medium (DMEM), fetal bovine serum (FBS), Roswell Park Memorial Institute Medium (RPMI), and 2,3-bis[2-methoxy-4-nitro-5-sulfophenyl]-2*H*-tetrazolium-5-carboxyanilide inner salt (XTT) were obtained from Life Technologies.

General characterization

(1) Scanning electron microscopy (SEM) images were obtained using an FEI Teneo VolumeScope with an operation voltage of 10 kV.

(2) Transmission electron microscopy (TEM) images and energy-dispersive X-ray spectroscopy (EDX) profiles were acquired using an FEI Tecnai F20 instrument with an operation voltage of 200 kV.

(3) Atomic force microscopy (AFM) experiments were carried out with an Asylum Research Cypher ES atomic force microscope. Typical scans were conducted in air in intermittent contact mode with silicon cantilevers (Budget Sensors Tap 300).

(4) Small-angle X-ray scattering (SAXS) data were collected at the SAXS beamline of the Australian Synchrotron facility, part of the Australian Nuclear Science and Technology

Organisation (ANSTO). The samples were investigated using the small/wide-angle X-ray scattering beamline (16 keV, 7000 mm camera length using Pilatus 1M and 200K detectors, transmission mode). Scatterbrain software was used for the analysis.

(5) Hydrodynamic sizes and zeta potential were measured using dynamic light scattering (DLS) with a Malvern NANO-ZS90 Zetasizer.

(6) Nuclear Magnetic Resonance (NMR) spectroscopy was conducted on a Varian Unity 400 MHz spectrometer at room temperature. Samples were dissolved in 0.5 mL DMSO-d6.

(7) UV–visible absorption measurements were performed on an Analytik Jena SPECORD 250 PL instrument.

(8) X-ray powder diffraction (XRD) was performed on a Bruker D8 Advance diffractometer. Stepsize was set as 0.02.

(9) X-ray photoelectron spectroscopy (XPS) was performed on a VG ESCALAB220i-XL spectrometer equipped with a hemispherical analyzer. The incident radiation was monochromatic Al Kα X-rays (1486.6 eV) at 220 W (22 mA and 10 kV). The data were processed using CasaXPS software, and the energy calibration was referenced to the C 1s peak at 285.0 eV. For sample preparation, the particles were prepared on planar silicon substrates and allowed to air dry.

(10) Fourier transform infrared (FTIR) spectroscopy was performed on a Bruker Tensor II FTIR spectrophotometer

(11) Thermogravimetric analysis (TGA)–FTIR spectroscopy was performed on a Netzsch TG 209 instrument coupled with a Bruker Alpha transmission FTIR module. Data were processed using the Netzsch Proteus software.

(12) Dark-field images were collected using an Olympus 71 inverted microscope with an oil-immersion dark-field condenser at 100× magnification.

Cytotoxicity study of the pBDT-TA particles

Raw cell viability by the cell proliferation kit II (XTT) assay: Raw cells were seeded in a 96-well plate (Costar, Corning, USA) at a density of 5,000 cells per well in 100 μL DMEM supplied with 10% FBS for 24 h. Then, the culture media was removed and replaced with fresh media (for cell control) or fresh media containing the pBDT-TA particles (100 nm) at a particle concentration of 0, 25, 50, 100, 150, or 200 µg mL^{-1} for 48 h. After the treatment, the media was replaced with fresh media containing activated XTT (9 mL of 0.2 mg mL^{-1} XTT in complete DMEM could be activated by adding 22.5 μL of 0.6 mg mL^{-1} PMS in DPBS), and cells were incubated for 3 h. Finally, cells were screened on an Infinite M200 microplate reader (Tecan, Switzerland). Absorbance readings were measured at 475 nm and a reference wavelength of 675 nm was used. Cell viability was expressed as a percentage by normalizing the absorbance to untreated cells. All experiments were performed in triplicates, and data are presented as mean ± standard deviation.

THP-1 Cell viability by XTT assay: THP-1 cells were seeded in a 96-well plate (Costar, Corning, USA) at a density of 5,000 cells per well in 100 μL RPMI supplied with 10% FBS for 24 h. Then, pBDT-TA nanoparticles were directly injected to the cell at a particle concentration of 0, 25, 50, 100, 150, or 200 µg mL^{-1} for 48 h. After the treatment, 80 μL complete RPMI media containing activated XTT was introduced to each well to reach the final XTT concentration of 0.2 mg mL^{-1} (note: 13.5 µg PMS was required to activate 1.8 mg XTT), and cells were incubated for 3 h. Finally, cells were screened on an Infinite M200 microplate reader. Absorbance readings were measured at 475 nm and a reference wavelength of 675 nm was used. Cell viability was expressed

as a percentage by normalizing the absorbance to untreated cells. All experiments were performed in triplicates, and data are presented as mean ± standard deviation.

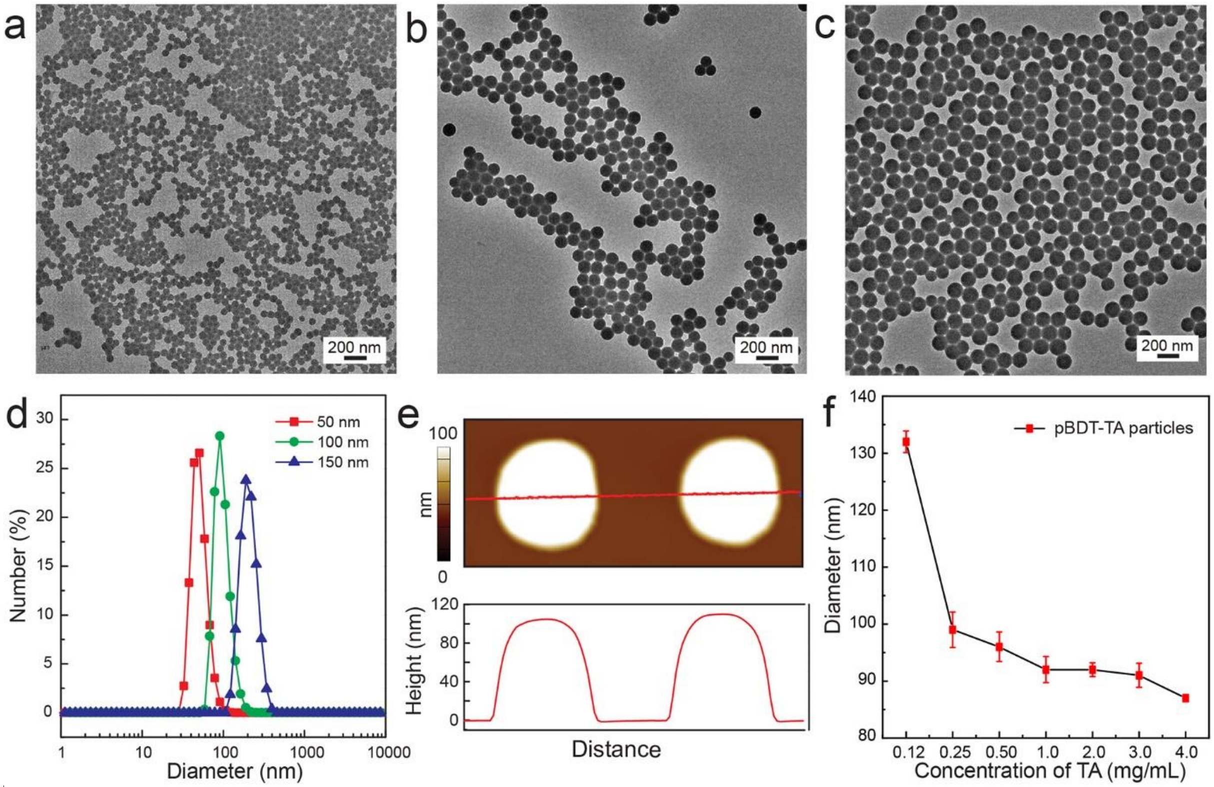

Supplementary Fig. 1. Characterization of pBDT-TA particles of different diameters.

TEM images of pBDT-TA particles with different diameters: (a) 50, (b) 100, and (c) 150 nm. (d) DLS results of the pBDT-TA particles with different diameters. The concentration of BDT used to engineer 50, 100, and 100 nm particles was 0.25, 0.5 and 1.0 mg mL^{-1}, respectively, while the concentration of TA was fixed at 0.5 mg mL^{-1}. (e) Representative AFM height image and corresponding line profile of 100 nm pBDT-TA particles. (f) Size of pBDT-TA particles prepared using different TA concentrations. The concentration of BDT was fixed at 0.5 mg mL^{-1}. The error bars represent standard deviations (n = 3).

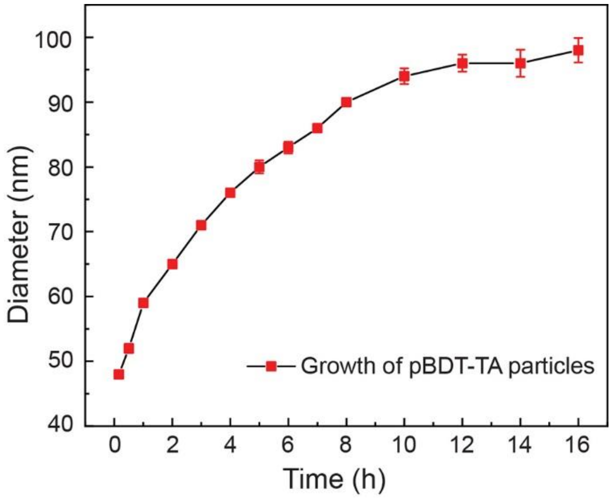

Supplementary Fig. 2. Kinetics of growth of pBDT-TA particles.

Size of pBDT-TA particles as a function of time during the assembly process, as measured by dynamic light scattering. The first time point was collected after mixing TA and BDT for 10 min. The error bars represent standard deviations (n = 3).

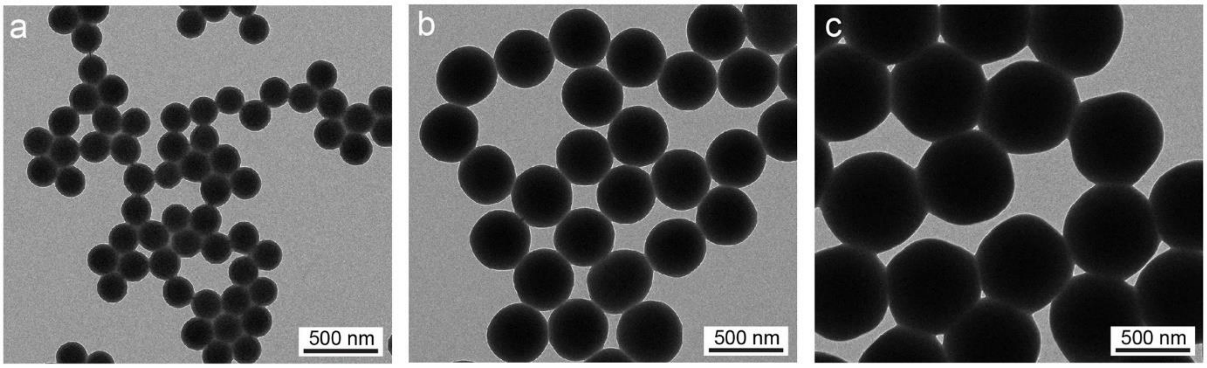

Supplementary Fig. 3. TEM analysis of large pBDT-TA particles.

TEM images of large pBDT-TA particles prepared by the seeded growth method: (a) 200, (b) 400, and (c) 650 nm.

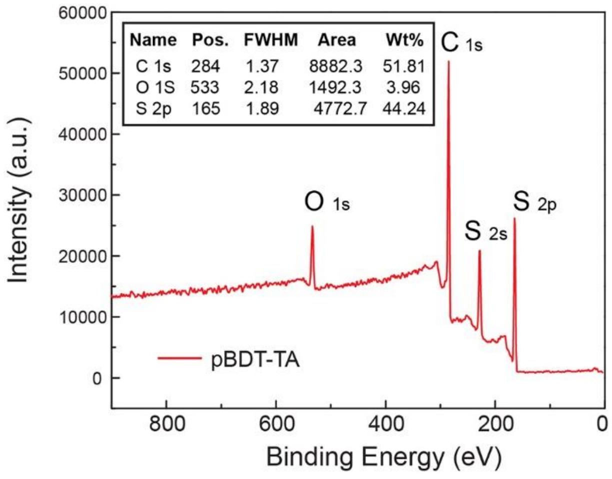

Supplementary Fig. 4. XPS analysis of pBDT-TA particles.

XPS wide scan of pBDT-TA particles. Inset: quantitative analysis of the pBDT-TA particles.

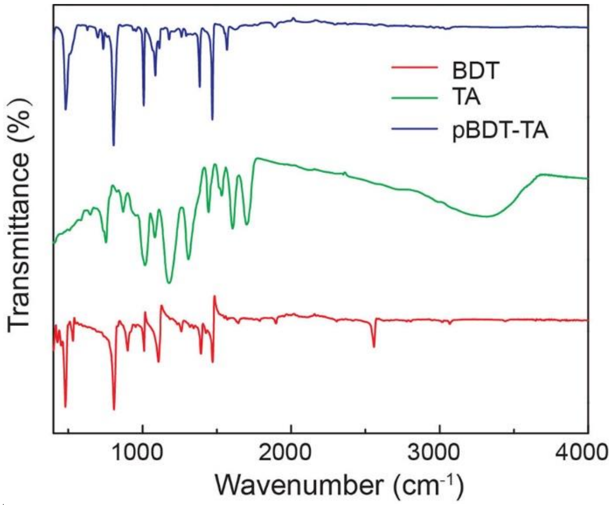

Supplementary Fig. 5. FTIR analysis of pBDT-TA particles.
FTIR spectra of BDT, TA, and pBDT-TA particles.

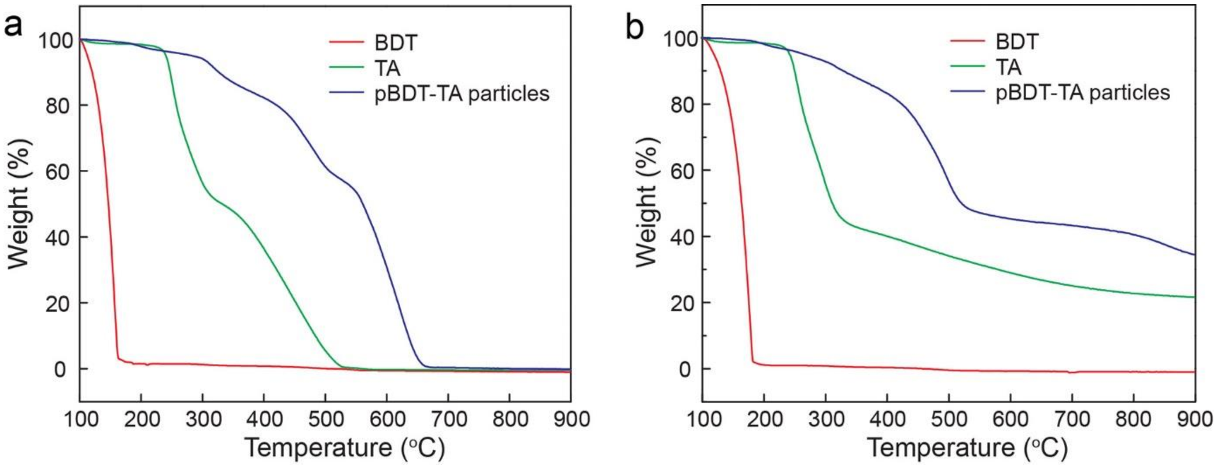

Supplementary Fig. 6. Thermal stability of pBDT-TA particles assessed by TGA.
TGA curves of BDT, TA, and pBDT-TA particles obtained under (a) air and (b) nitrogen.

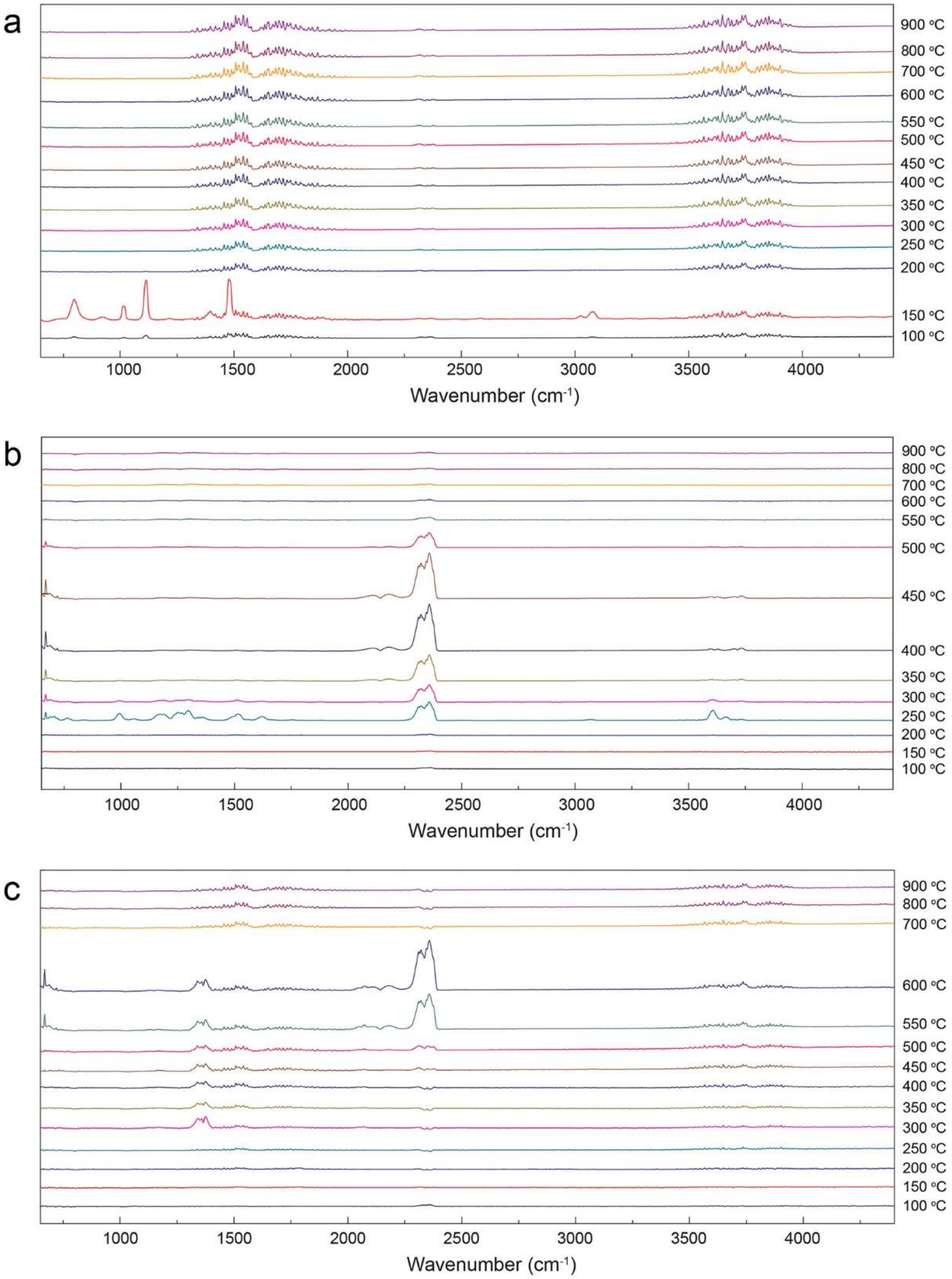

Supplementary Fig. 7. Thermal stability of pBDT-TA particles assessed by XRD.

TGA–FTIR results of (a) BDT, (b) TA, and (c) pBDT-TA particles obtained under air. BDT molecules volatilize completely before 200 °C.

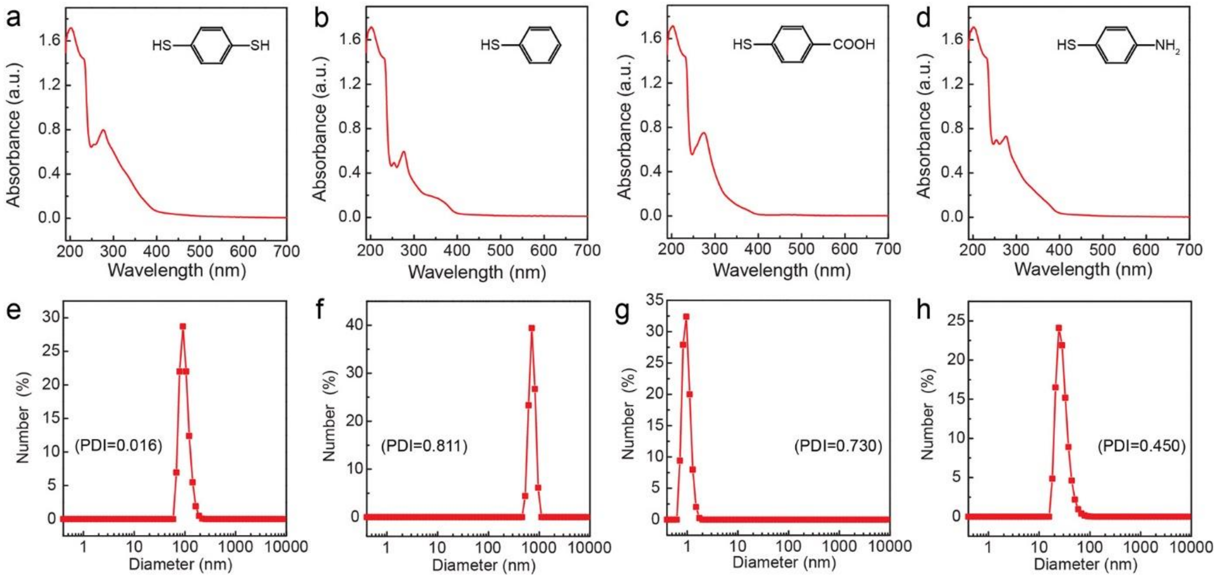

Supplementary Fig. 8. Study of complex formation from different types of thiol-carrying benzene molecules.

(a–d) UV–vis spectra of the reaction solutions after 12 h in the presence of different thiol-containing benzene molecules i.e., BDT (a), thiophenol (b), 4-mercaptobenzoic acid (c), and 4-aminothiophenol (d) and (e–h) the corresponding DLS results of the complexes. The large polydispersity index values in (f–h) indicate the broad size distribution of complexes in the final products.

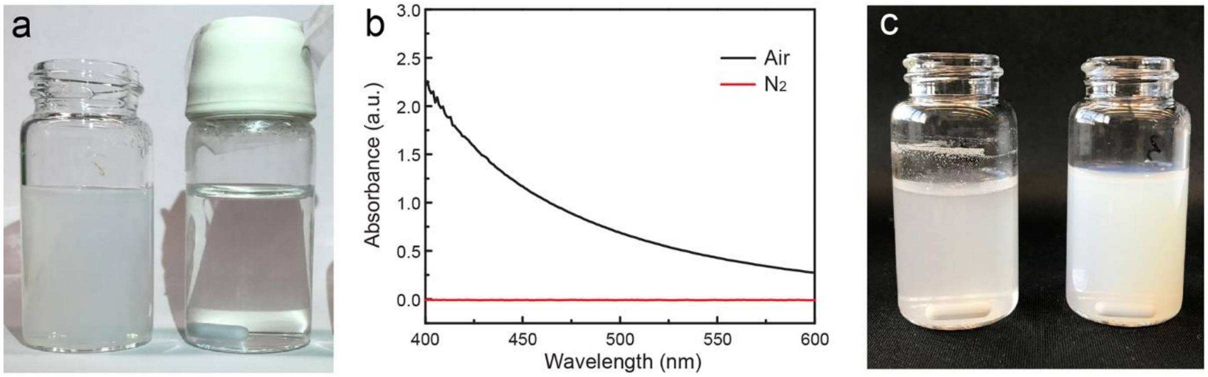

Supplementary Fig. 9. Polymerization reactions under mild conditions.

(a) Photograph of the products obtained from reactions performed in the presence (left) and absence (right) of air and (b) corresponding UV–vis spectra of the products. The reaction performed in the absence of air was achieved by purging the reaction solution with nitrogen to displace the air in the reaction system. The transparent solution obtained in the nitrogen-purged reaction indicates that the formation of particles requires oxygen. (c) Photograph of the products obtained from the polymerization of BDT in the absence (left) and presence (right) of TA.

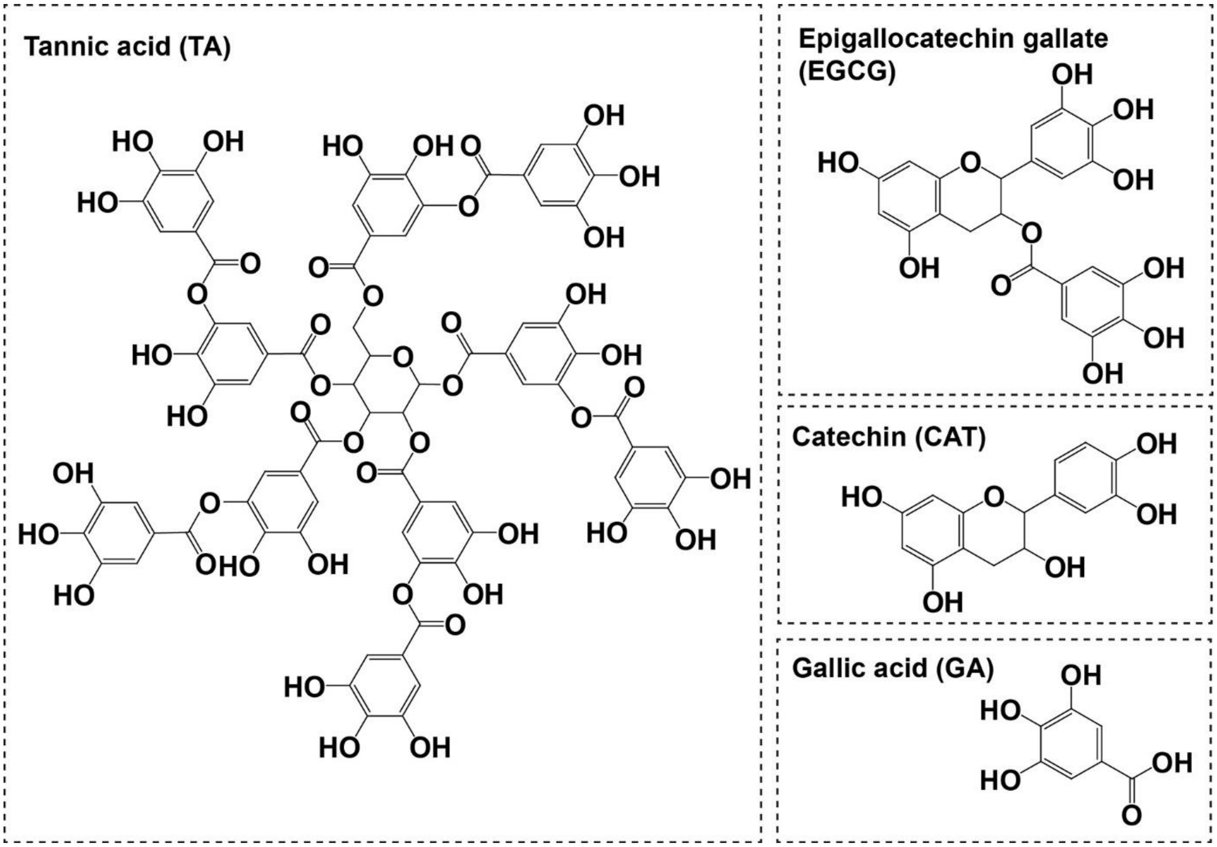

Supplementary Fig. 10. Chemical structures of polyphenols.
Chemical structures of the different polyphenols (TA, EGCG, CAT, GA) studied in this work.

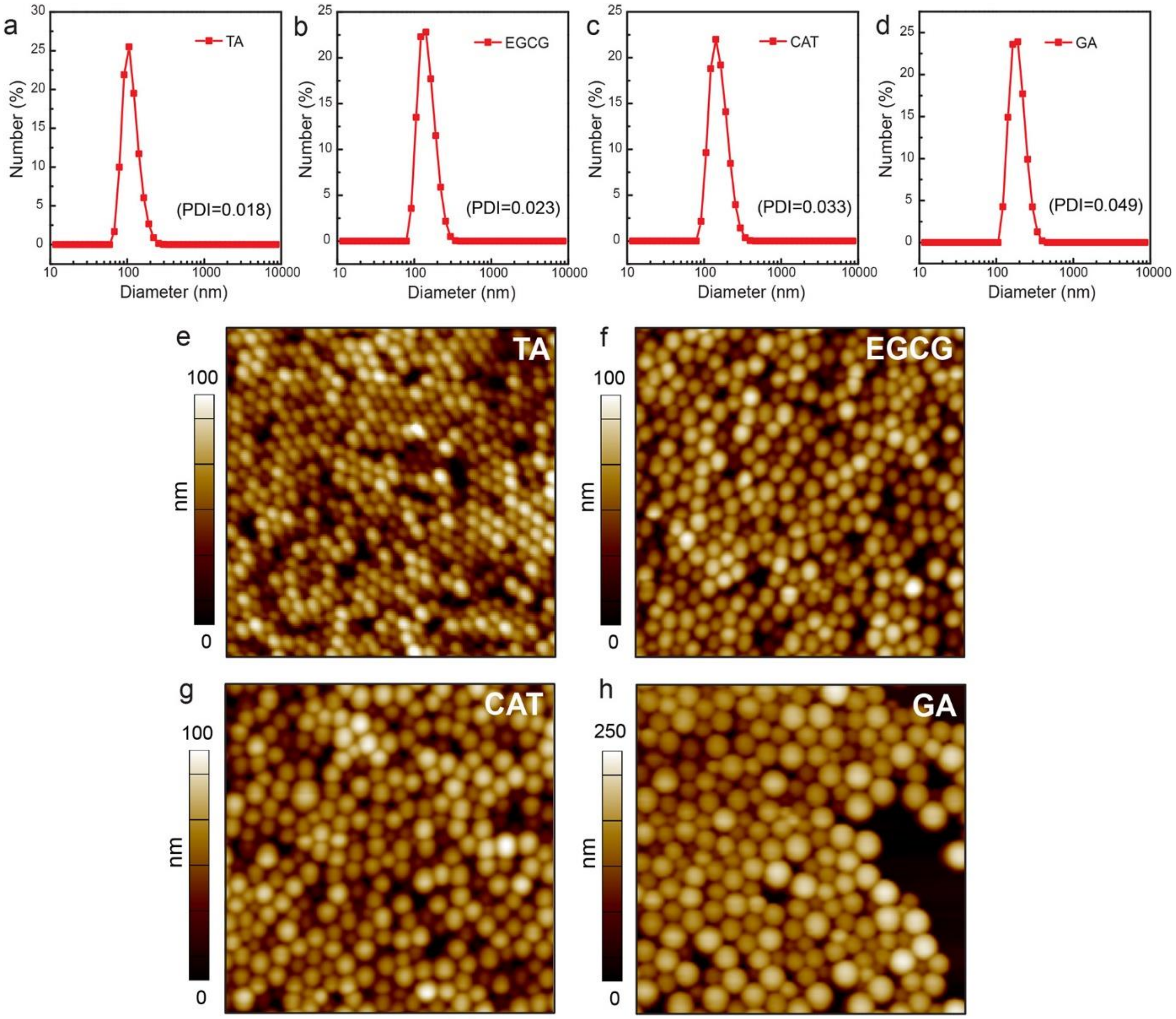

Supplementary Fig. 11. Particles prepared by using different polyphenols.

(a–d) DLS results and (e–h) corresponding AFM images of the particles using TA, EGCG, CAT, and GA.

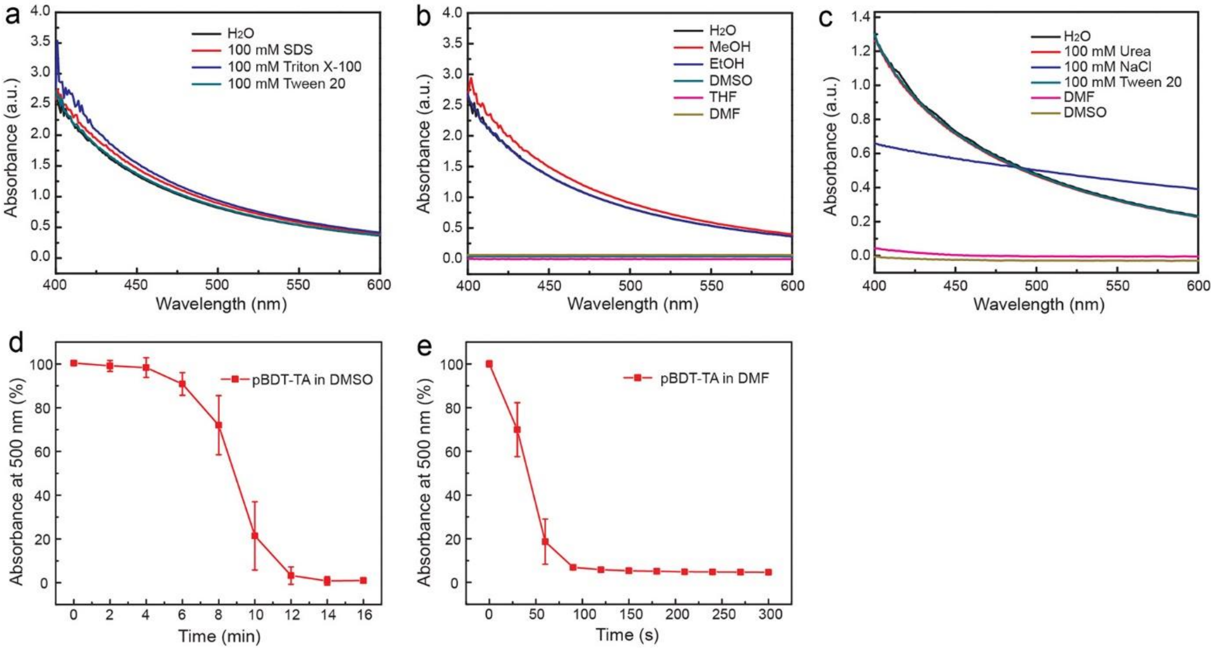

Supplementary Fig. 12. Stability of pBDT-TA particles in different solvents.

UV–vis spectra of the pBDT-TA particles in different (a) aqueous environments and (b) organic solvents. (c) UV–vis spectra of the pBDT-TA particles in different media to investigate the interaction in the complexation. Disassembly of the pBDT-TA particles in (d) DMSO and (e) DMF; the error bars represent standard deviations (n = 3).

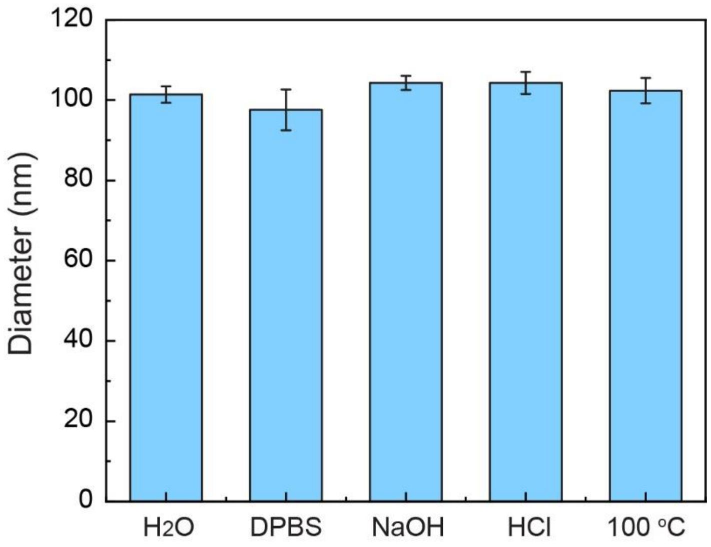

Supplementary Fig. 13. Stability of pBDT-TA particles in different environments.

Stability of pBDT-TA particles in different aqueous environments after 1 h of incubation (the error bars represent standard deviations, n = 3). The concentrations of NaOH and HCl were 100 mM (corresponding to pH 13 and pH 1, respectively).

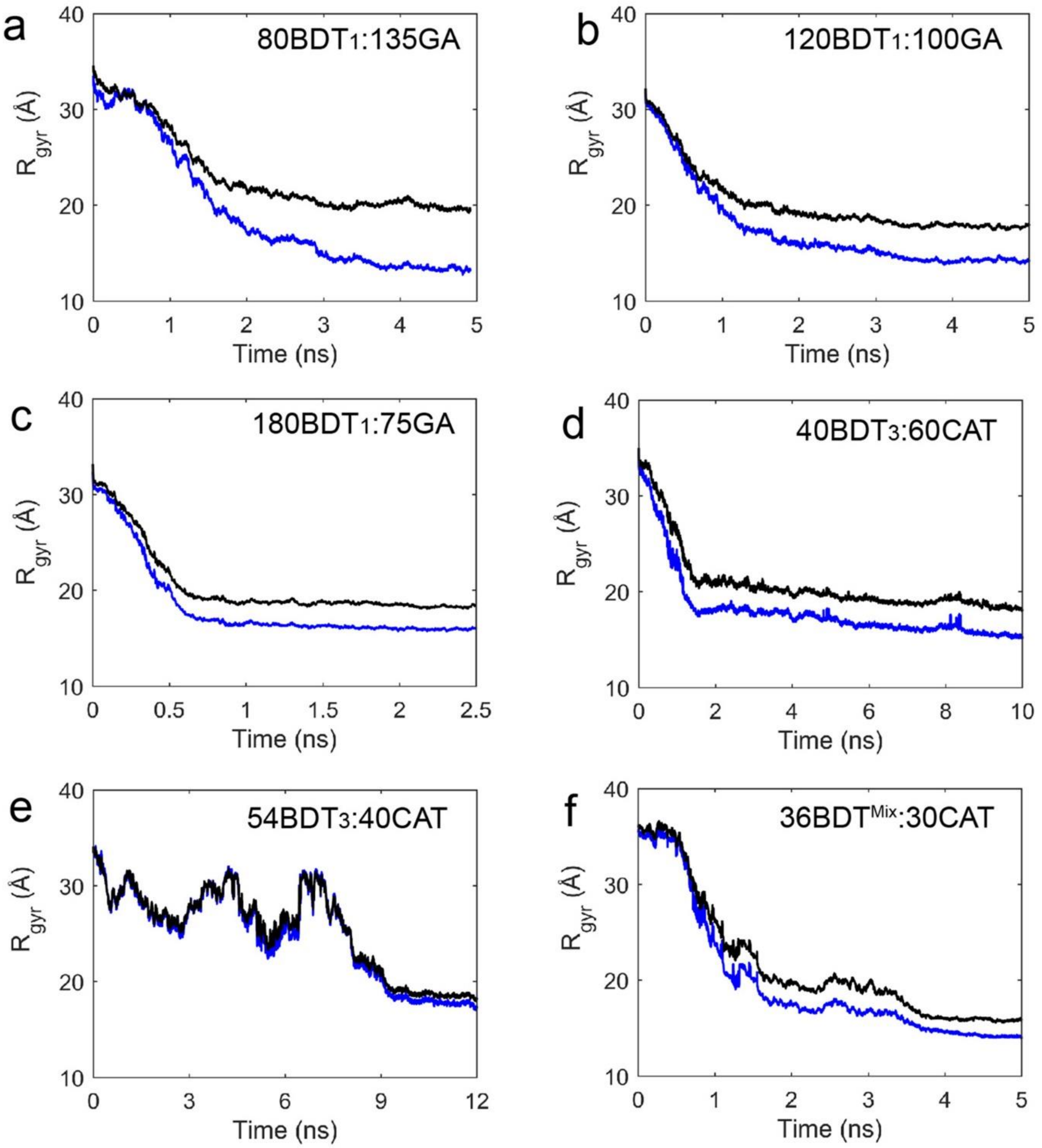

Supplementary Fig. 14. MD simulation of the assembly of BDT and polyphenols.

MD simulation of the assembly under different BDT/polyphenol (GA or CAT) ratios in water: (a) 80:135, (b) 120:100, (c) 180:75, (d) 40:60, (e) 54:40, and (f) 36:30. Radius of gyration (R_{gyr}) of pBDT (blue) and pBDT-polyphenols (black).

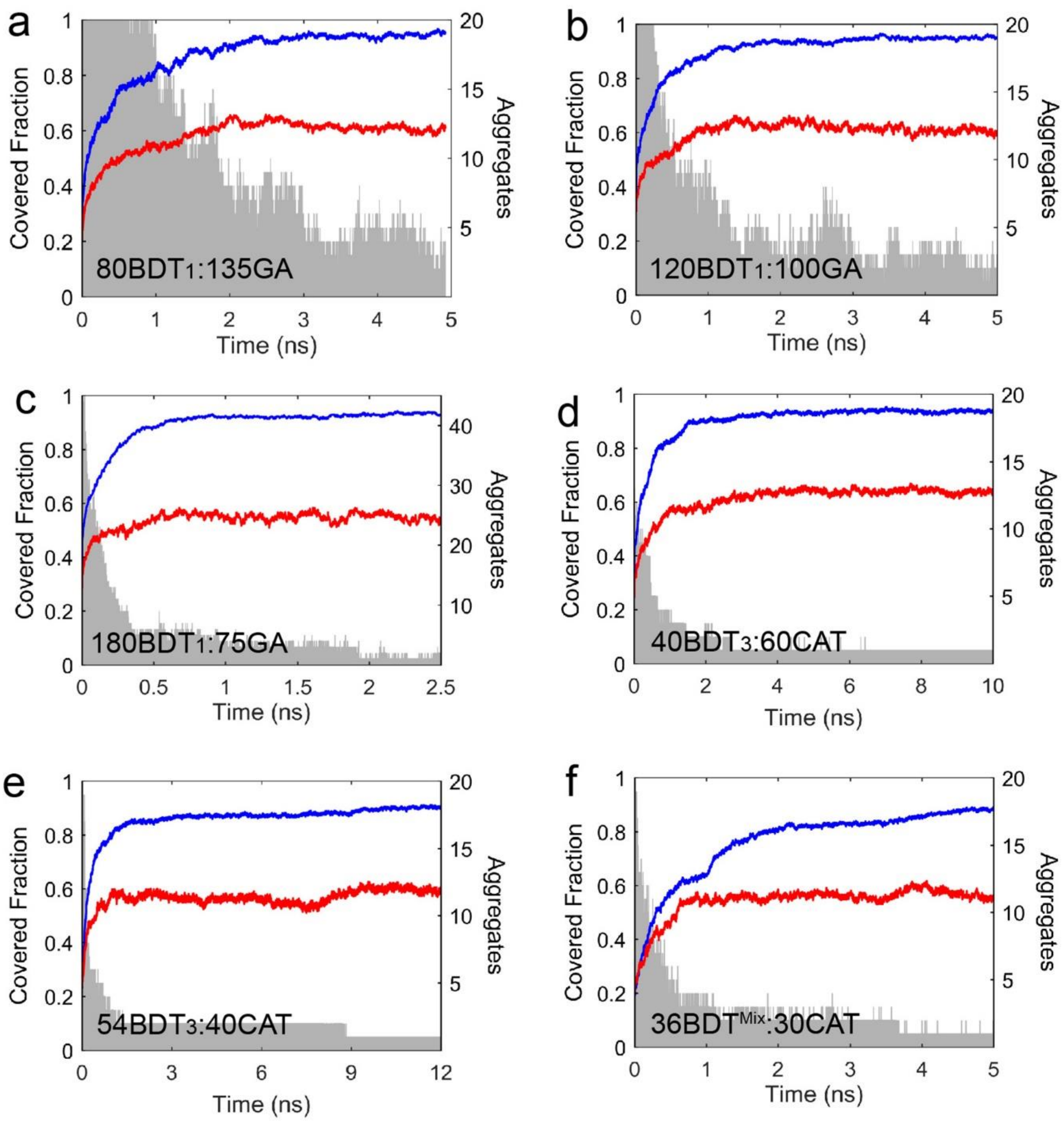

Supplementary Fig. 15. MD simulation of the assembly of BDT and polyphenols.

MD simulation of the assembly under different BDT/polyphenol (GA or CAT) ratios in water: (a) 80;135, (b) 120:100, (c) 180:75, (d) 40:60, (e) 54:40, and (f) 36:30. Covered fraction of pBDT (blue) and GA/CAT (red) molecules (i.e., not exposed to solvent owing to proximity to other BDT or polyphenol molecules), and number of pBDT aggregates (gray).

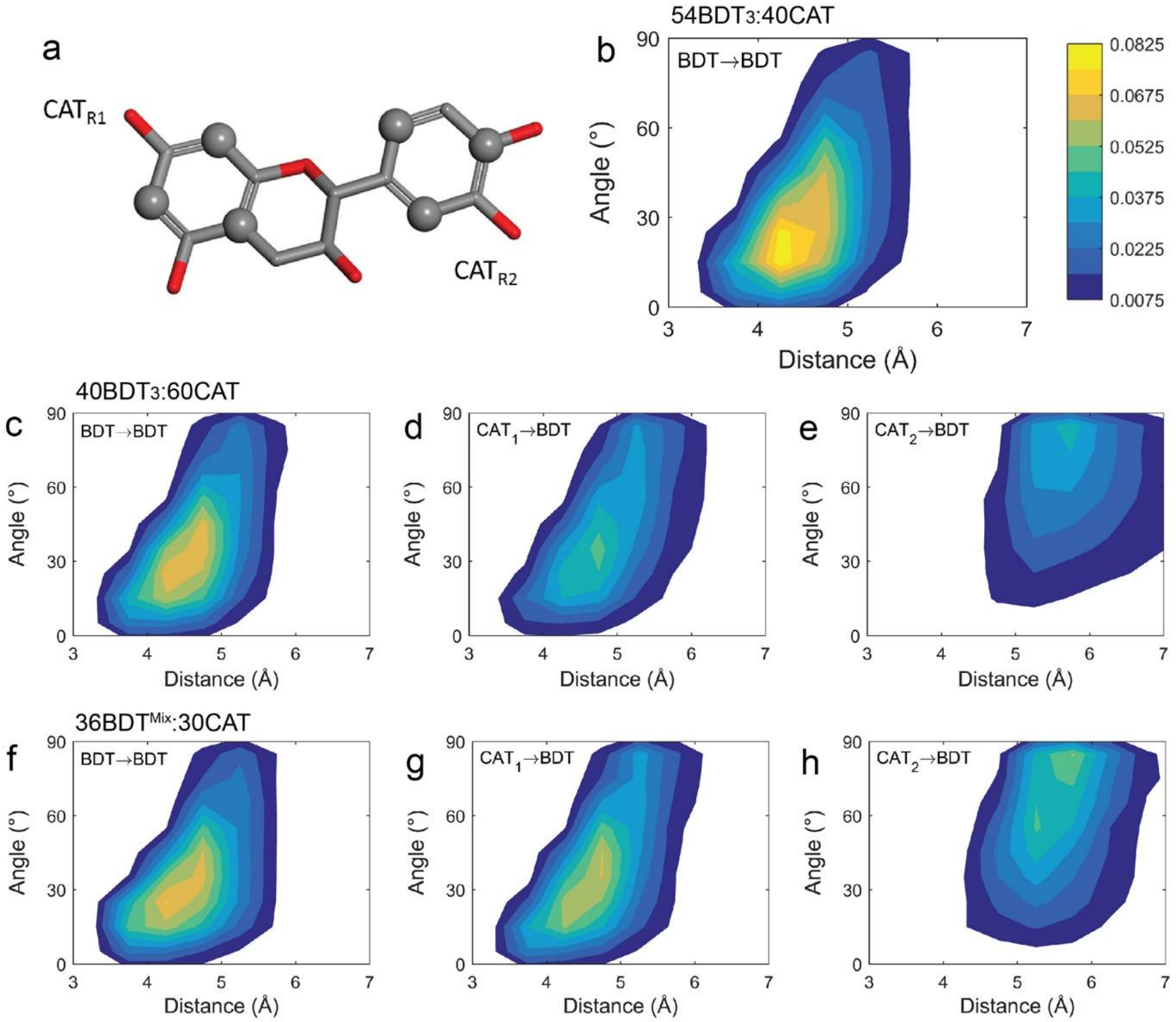

Supplementary Fig. 16. Population distribution of the angle between different aromatic rings.

(a) Molecular structure of catechin (CAT) showing atoms used to define aromatic ring planes as filled balls; hydrogen atoms are not shown for clarity. (b) Population distribution of the angle between the plane of the aromatic group of each BDT ring and the nearest BDT aromatic group against distance between the two rings. (c–h) Angle against distance population distributions for BDT aromatic rings to the nearest BDT aromatic ring (c, f), angle against distance population distributions for CAT_1 aromatic rings to the nearest BDT aromatic ring (d, g), and angle against distance population distributions for CAT_2 aromatic rings to the nearest BDT aromatic ring (e, h) in different complexes prepared by different precursor ratios, i.e., 40BDT$_3$:60CAT (c–e) and 36BDT^{Mix}:30CAT (f–h).

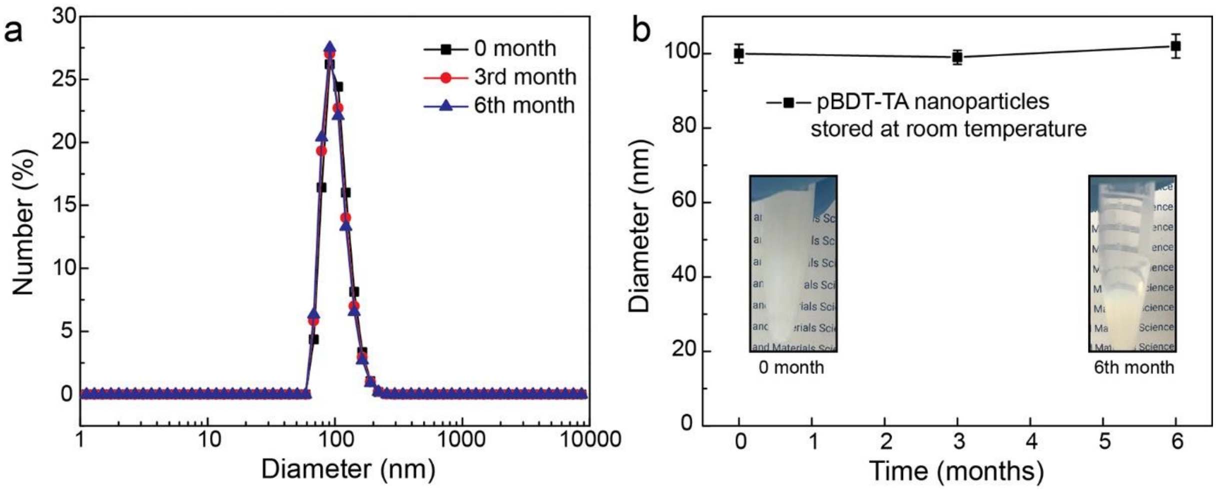

Supplementary Fig. 17. Long-term stability of pBDT-TA particles.

Long-term stability of pBDT-TA particles (100 nm; 5 mg mL^{-1}) stored at room temperature. (a) DLS results of the pBDT-TA particles at different time points. (b) Changes in the diameter of the pBDT-TA particles as a function of time. Insets: photographs of the solutions at 0 and 6 months (the error bars represent standard deviations, $n = 3$).

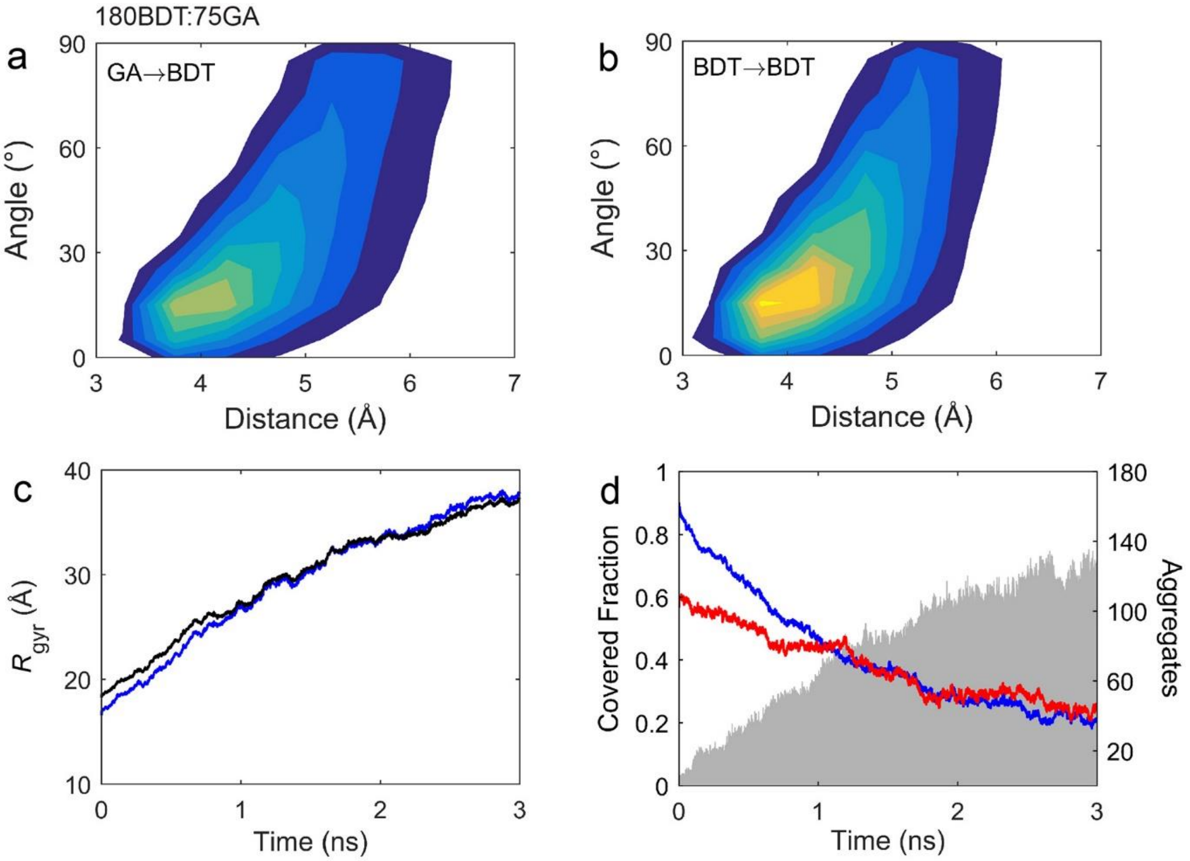

Supplementary Fig. 18. MD simulation of the assembly of BDT and GA.

(a) Angle against distance population distributions for GA aromatic rings to the nearest BDT aromatic ring. (b) Angle against distance population distributions for BDT aromatic rings to the nearest BDT aromatic ring. (c, d) Disassembly process of BDT-GA complexes in DMSO: R_{gyr} of BDT (blue) and BDT-GA (black) in DMSO (c) and covered fraction of BDT (blue) and GA (red) molecules (i.e., not exposed to solvent owing to proximity to other BDT or GA molecules) and number of BDT aggregates (gray) (d).

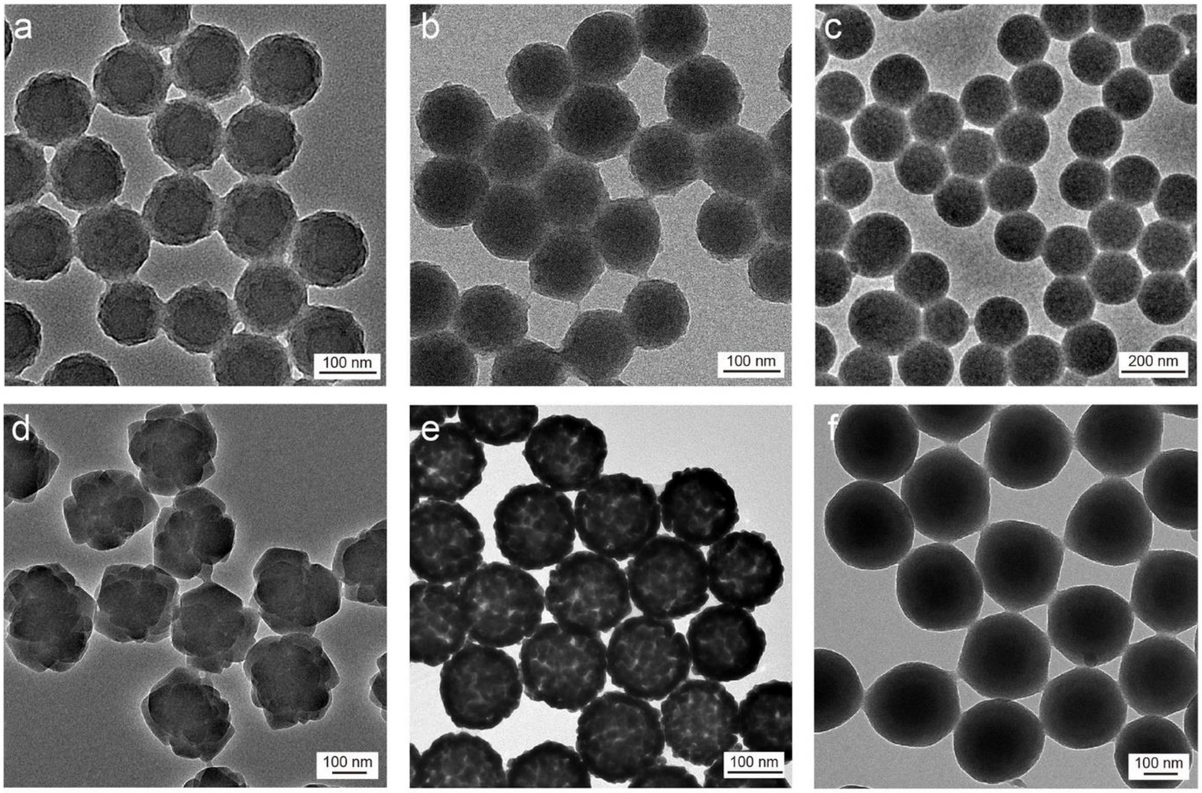

Supplementary Fig. 19. pBDT-TA particles with different shell materials.

TEM images of pBDT-TA particles with different shell materials: (a) polydopamine, (b) polypyrrole, (c) MPN (i.e., Fe^{III}-TA), (d) metal–organic framework (i.e., ZIF-8), (e) gold, and (f) silica.

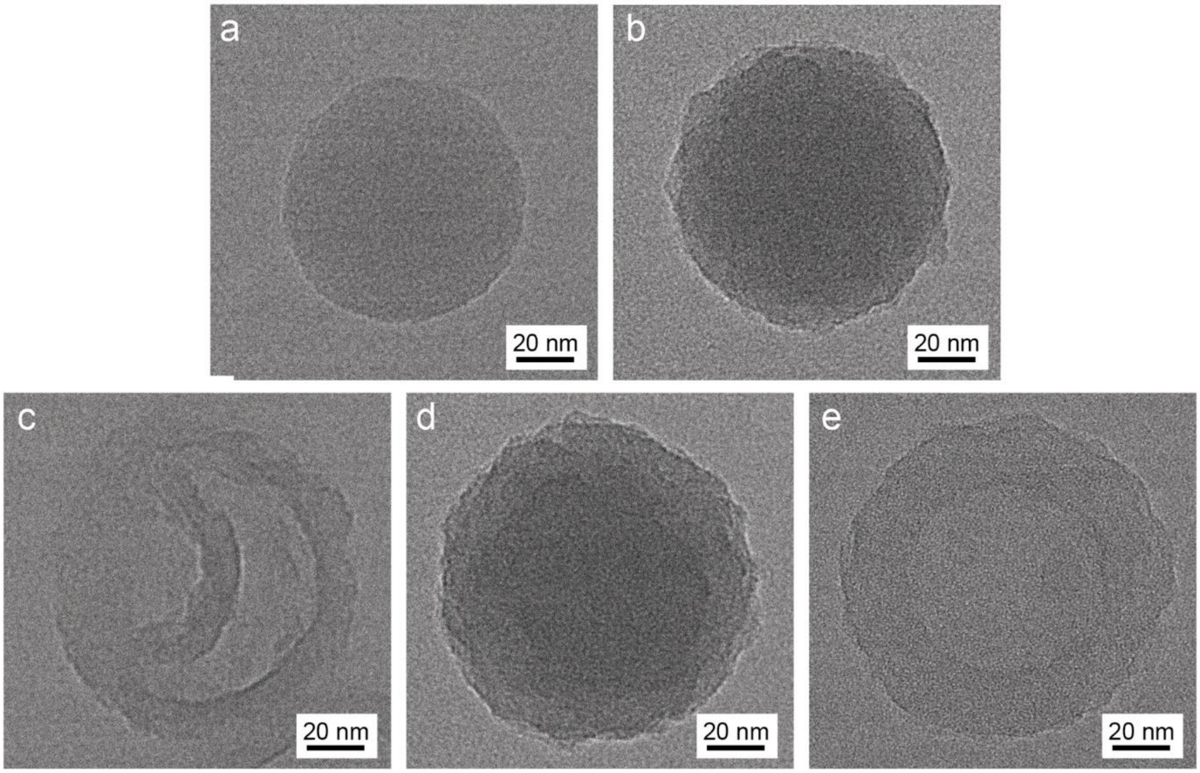

Supplementary Fig. 20. pBDT-TA particles with PDA shells of different thicknesses.

TEM images of pBDT-TA nanoparticles with PDA shells of different thicknesses (15 and 25 nm by using 0.1 and 0.6 mg mL^{-1} dopamine, respectively) before and after core removal: (a) pBDT-TA particles, (b) pBDT-TA@PDA15 nm, (c) pBDT-TA@PDA15 nm after removal of the core, (d) pBDT-TA@PDA25 nm, and (e) pBDT-TA@PDA25 nm after removal of the core. The clear hollow structure indicates the successful removal of the interior complexes.

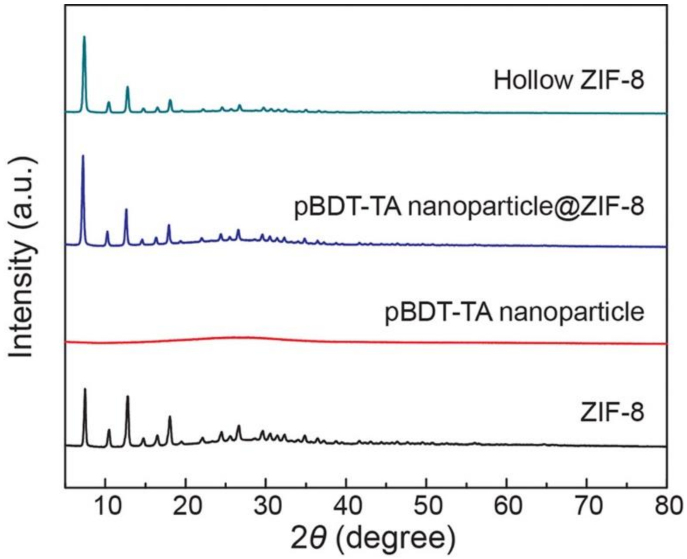

Supplementary Fig. 21. XRD analysis of ZIF-8-coated pBDT-TA particles.

XRD patterns of pure nanoscale ZIF-8 particles (prepared according to a previous report[1]), pBDT-TA particles, pBDT-TA particles coated by ZIF-8, and hollow ZIF-8 after removal of the pBDT-TA particles in the core.

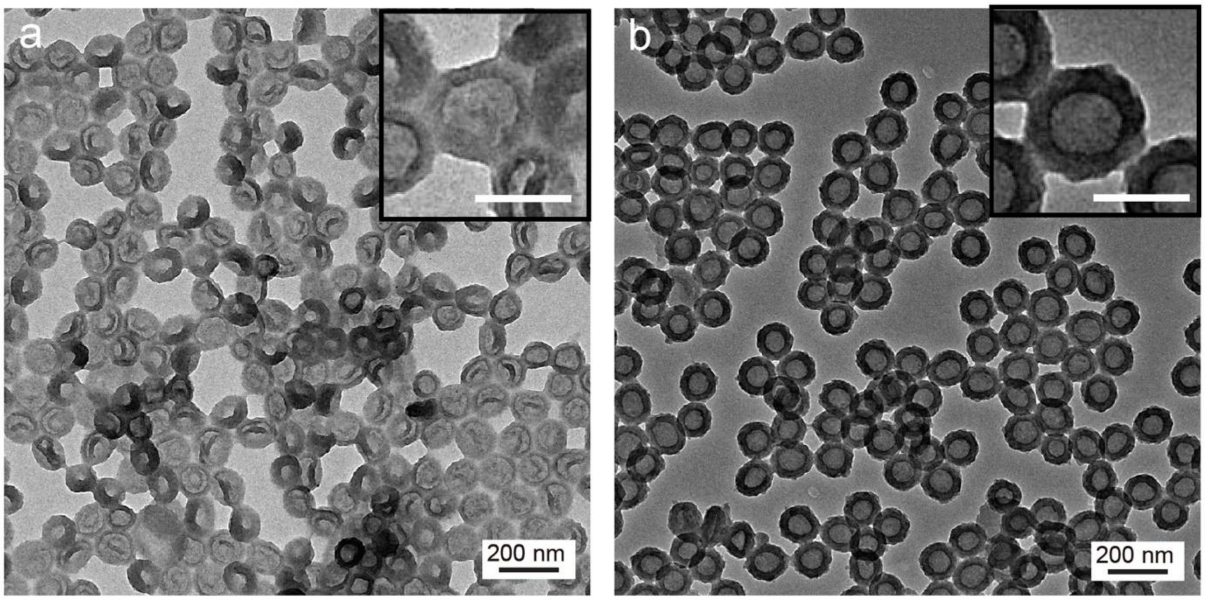

Supplementary Fig. 22. Control of shell thickness.

TEM images of PDA capsules with different shell thicknesses: (a) 15 and (b) 25 nm by using 0.1 and 0.6 mg mL^{-1} dopamine, respectively. Insets show higher magnification images of representative capsules (scale bar = 100 nm).

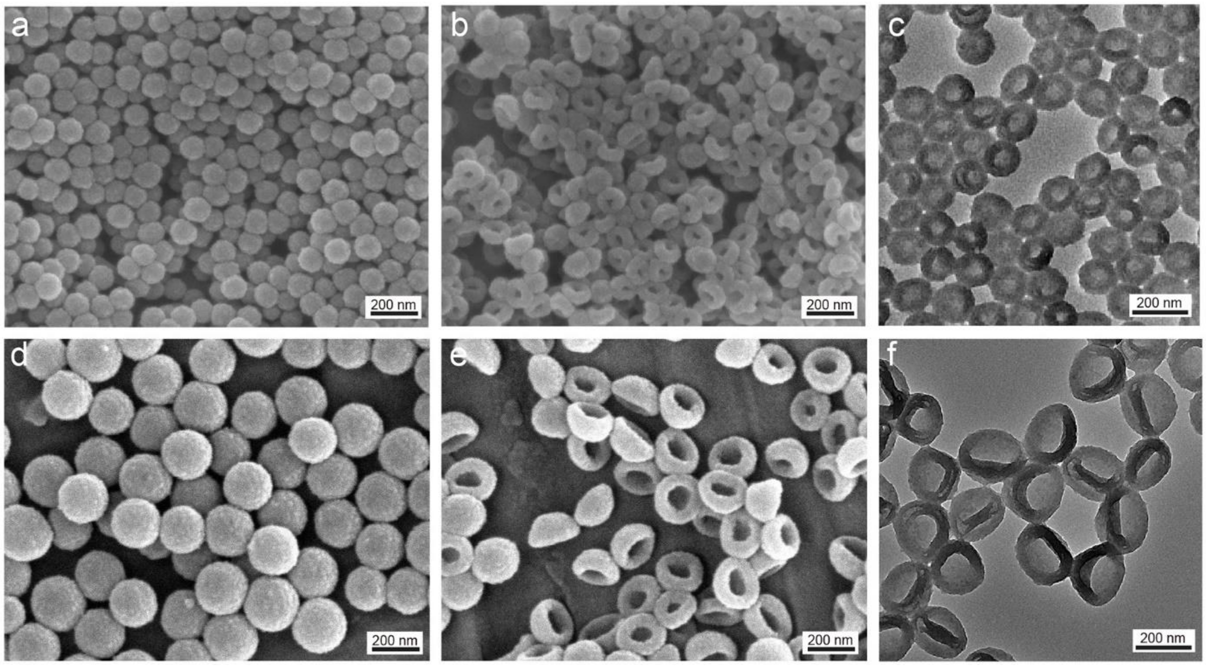

Supplementary Fig. 23. Control of inner particle diameter (cavity).

(a–f) SEM and TEM images of PDA capsules using small (100 nm) (a–c) and large (200 nm) (d–f) pBDT-TA particles. SEM images of (a, d) pBDT-TA@PDA and (b, e) pBDT-TA@PDA after removal of the core, and (c, f) TEM image of pBDT-TA@PDA after removal the core.

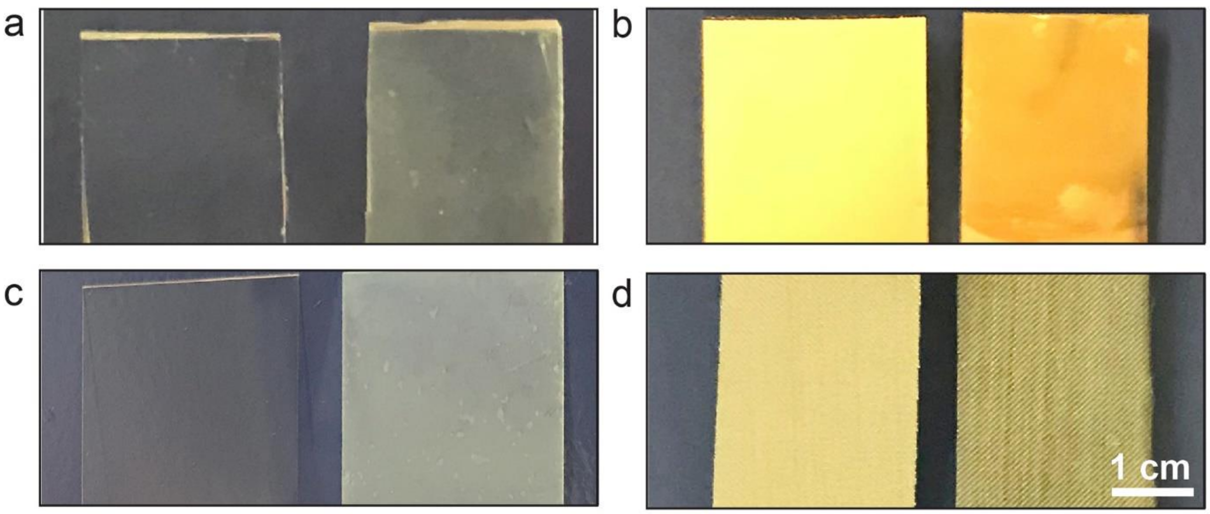

Supplementary Fig. 24. Coating of different substrates.

Photographs of various substrates before (left) and after coating (right) of pBDT-TA supramolecular complexes: (a) PDMS, (b) Au, (c) glass, and (d) stainless steel mesh.

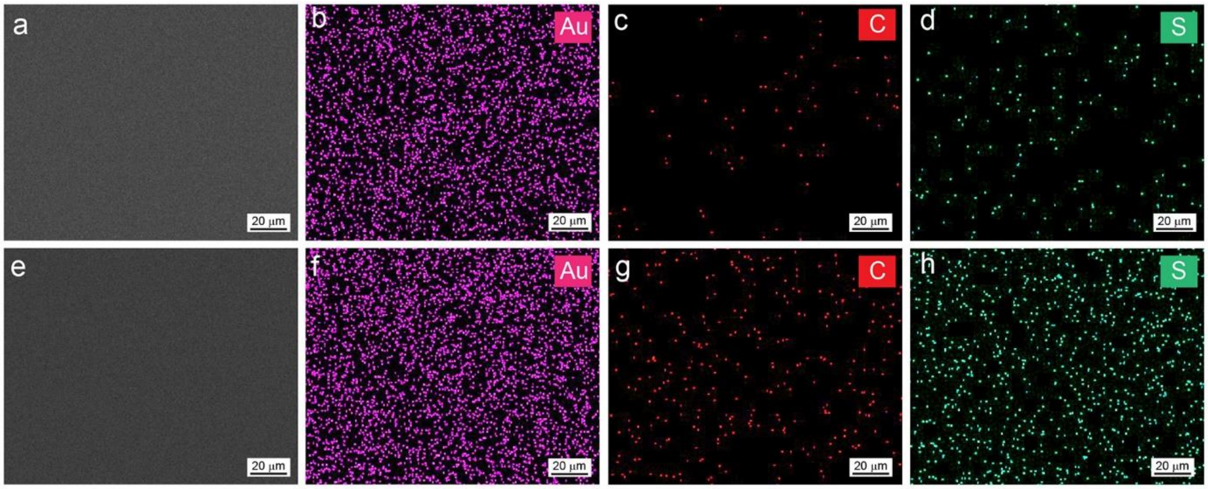

Supplementary Fig. 25. Coating of a Au substrate with pBDT-TA complexes.

SEM images of a (a) Au substrate and (e) Au substrate coated with pBDT-TA complexes. Elemental mapping of a (b–d) Au substrate and (f–h) Au substrate coated with pBDT-TA complexes.

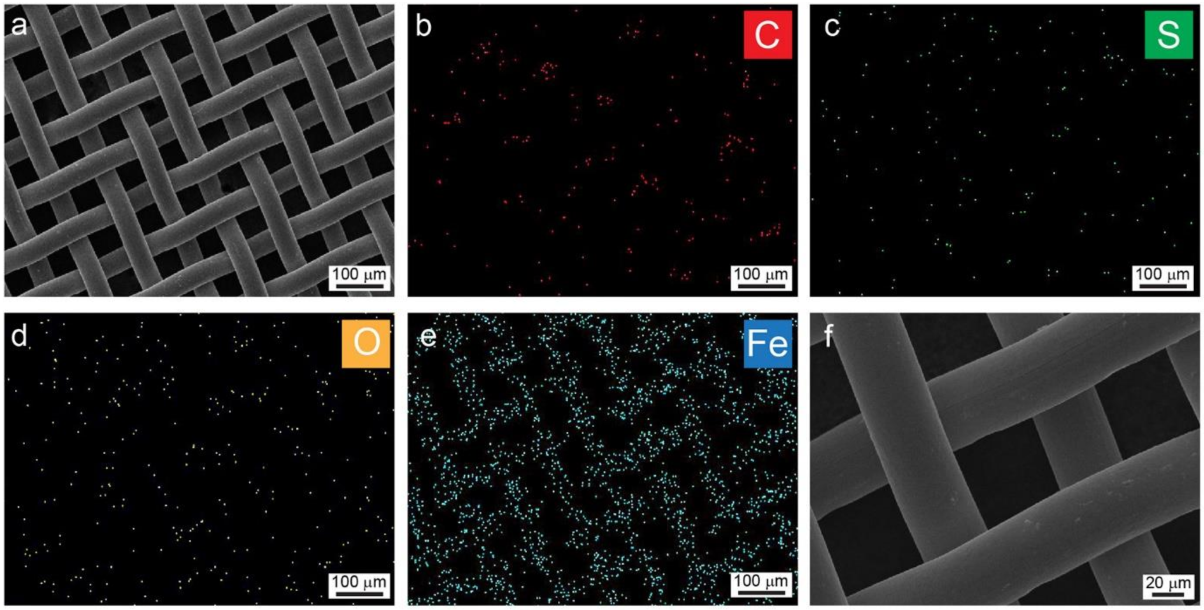

Supplementary Fig. 26. Surface analysis of a stainless steel wire mesh.

(a) SEM image of a stainless steel wire mesh. (b–e) Elemental mapping of C, S, O, and Fe. (f) SEM image of a stainless steel wire mesh at high magnification.

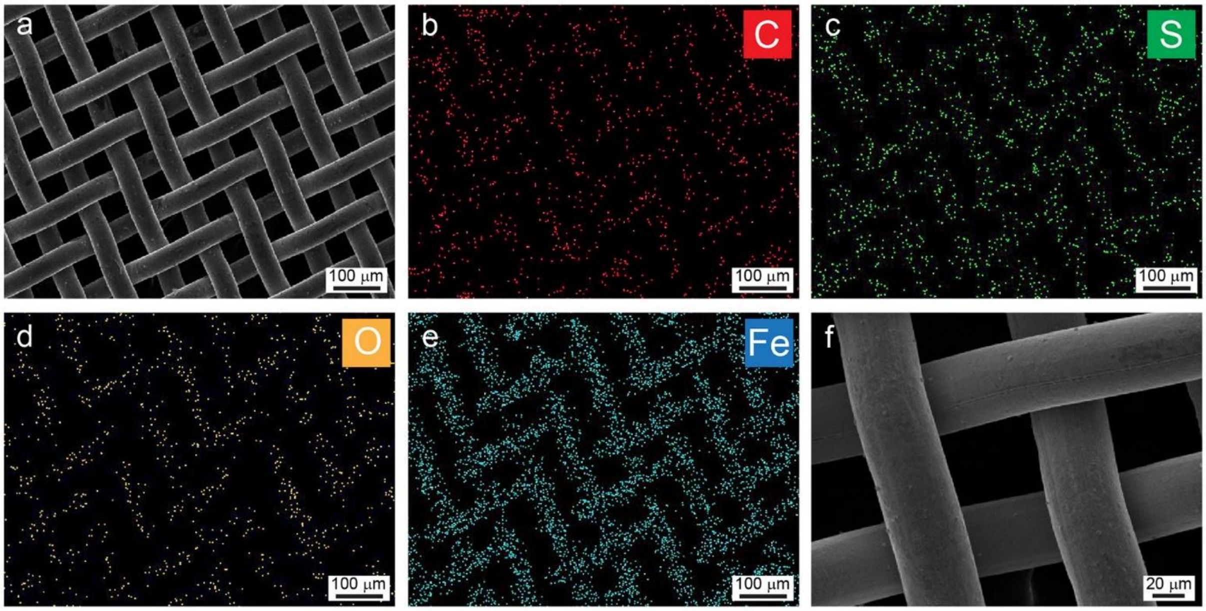

Supplementary Fig. 27. Coating of a stainless steel wire mesh with pBDT-TA complexes.

(a) SEM image of a stainless steel wire mesh coated by pBDT-TA complexes. (b–e) Elemental mapping of C, S, O, and Fe. (f) SEM image of a stainless steel wire mesh at high magnification.

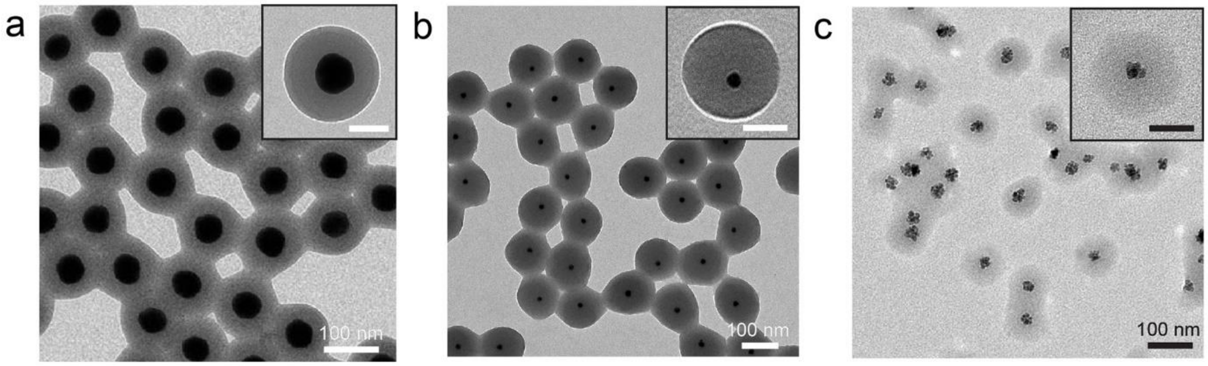

Supplementary Fig. 28. pBDT-TA coatings on different nanoparticles.

TEM images of particles of different sizes with pBDT-TA coating: (a) 50 nm Au nanoparticles (AuNPs), (b) 14 nm AuNPs, and (c) IO nanoparticles. Insets show higher magnification images of representative particles (scale bar = 50 nm).

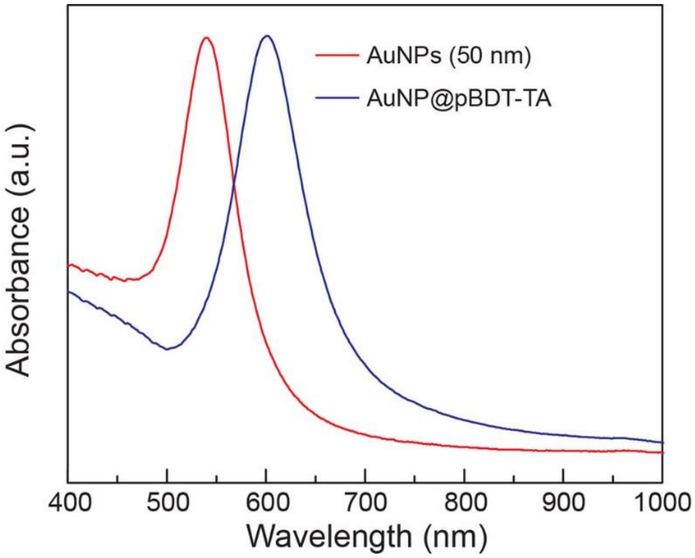

Supplementary Fig. 29. UV–vis spectroscopy of pBDT-TA-coated AuNPs.

UV–vis spectra of AuNPs (50 nm) and AuNP@pBDT-TA particles with a pBDT-TA shell of 40 nm. The red-shifted localized surface plasmon resonance of AuNP@pBDT-TA indicates pBDT-TA coating on the AuNPs.

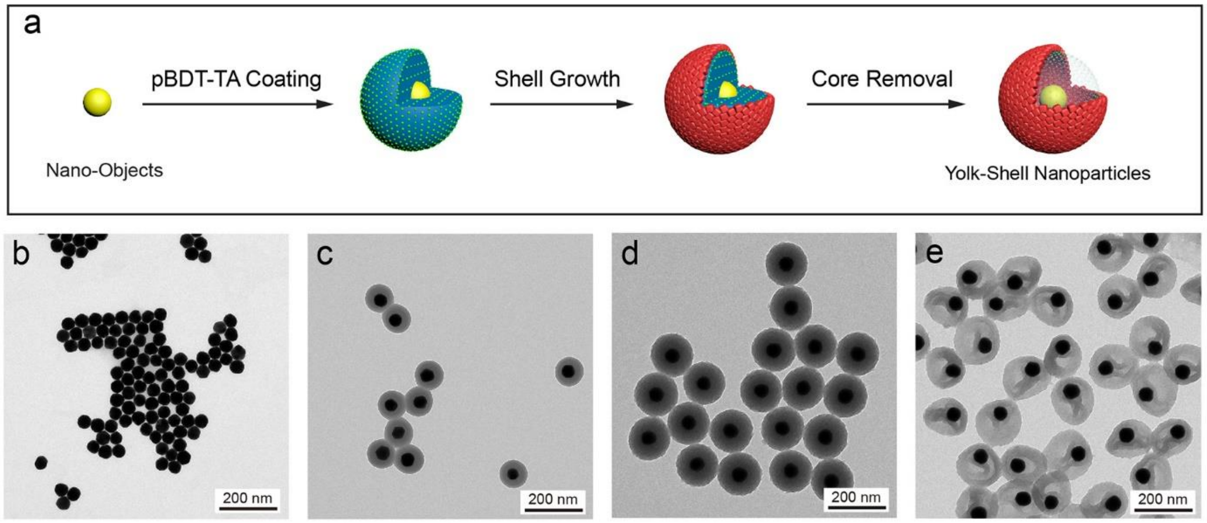

Supplementary Fig. 30. Synthesis of yolk–shell nanoparticles.

(a) Schematic of the synthesis of yolk–shell nanostructures using pBDT-TA complexes and corresponding TEM images of the different particles formed: (b) AuNPs, (c) AuNP@pBDT-TA, (d) AuNP@pBDT-TA@PDA, and (e) AuNP@PDA yolk–shell nanostructures.

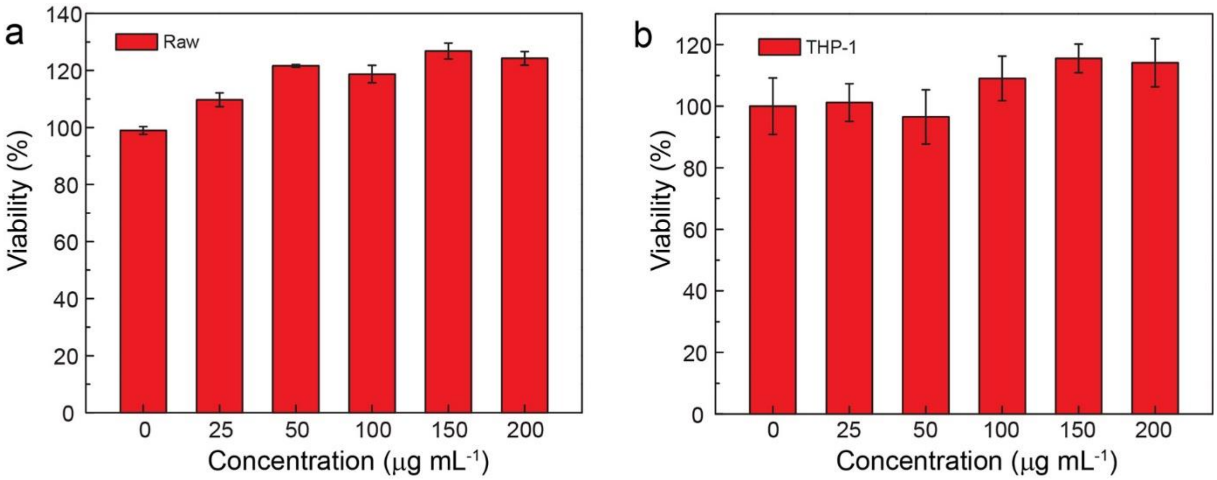

Supplementary Fig. 31. Cytotoxicity of pBDT-TA particles.

Cytotoxicity results of pBDT-TA particles (100 nm) for different cell lines: (a) Raw cells and (b) THP-1 cells (the error bars represent standard deviations, *n* = 3).

Supplementary Table 1.

Summary of the properties of the polyphenol-based particles prepared using different polyphenol species.

Particle	Polyphenol	Molecular weight (Da)	Size (nm)	ζ-Potential (mV)
pBDT-TA	TA	1,701	108.2 ± 1.4	−33.9 ± 0.3
pBDT-EGCG	EGCG	458	145.6 ± 3.6	−31.5 ± 0.8
pBDT-CAT	CAT	290	162.1 ± 1.7	−37.5 ± 0.6
pBDT-GA	GA	170	181.5 ± 2.7	−34.3 ± 0.5

TA, Tannic acid; EGCG, epigallocatechin gallate; CAT, catechin; GA, gallic acid.

Supplementary Table 2.

Model composition, dimensions and running times for the molecular dynamics simulations.

System	# of BDT			# of Polyphenol		Solvent	CD (g cm^{-3})	*D* (nm^3)	Time (ns)
	BDT$_1$	BDT$_3$	BDT$_6$	GA	CAT				
GA	-	-	-	1	-	H_2O	-	3.2	1.1
CAT	-	-	-	-	1	H_2O	-	3.2	1.1
80BDT$_1$:135GA	80	-	-	135	-	H_2O	0.15	7.2	5
120BDT$_1$:100GA	120	-	-	100	-	H_2O	0.15	7.2	5
180BDT$_1$:75GA	180	-	-	75	-	H_2O	0.15	7.2	2.5[*1]
							0.09	8.9	5
40BDT$_3$:60CAT	-	40	-	-	60	H_2O	0.15	7.2	10
									10
54BDT$_3$:40CAT	-	54	-	-	40	H_2O	0.15	7.2	10[*2]
									12
36BDT^{Mix}:30CAT	12	12	12	-	30	H_2O	0.10	7.5	7.5[*3]
									5
180BDT$_1$:75GA:DMSO	180	-	-	75	-	DMSO	-	7.8	3.0[a]
54BDT$_3$:40CAT:DMF	-	54	-	-	40	DMF	-	7.6	10.0[b]
									10.0[c]
36BDT^{Mix}:30CAT:DMF	12	12	12	-	30	DMF	-	7.6	10.0[d]
									10.0[e]

CD, Initial cell density of small molecules (ignoring water); *D*, Dimension of initial periodic box in $D \times D \times D$ nm^3; [a] Initial structure taken from (*1) at 2 ns; [b] Initial structure taken from (*2) at 9.5 ns; [c] Initial structure taken from (*2) at 10 ns; [d] Initial structure taken from (*3) at 7 ns; [e] Initial structure taken from (*3) at 7.5 ns.

Supplementary Table 3.

Average hydrogen bond data for systems containing catechin (CAT) solvated with H_2O.[(a)]

System	Hydrogen bonds (average per CAT)			
	CAT_{OH} with H_2O	CAT_O with H_2O	CAT_{OH} with CAT	CAT_O with CAT
CAT	2.96 ± 1.40	0.03 ± 0.16	-	-
$40BDT_3$:60CAT	2.07 ± 1.31	0.01 ± 0.08	0.50 ± 0.71	0.00 ± 0.05
$54BDT_3$:40CAT	2.30 ± 1.32	0.01 ± 0.08	0.28 ± 0.52	0.00 ± 0.03
$36BDT^{Mix}$:30CAT	2.39 ± 1.34	0.01 ± 0.08	0.23 ± 0.47	0.00 ± 0.01

(a) Based on geometric criteria for the presence of a hydrogen bond with a distance cutoff of 3.0 Å and an angle cutoff of 20°.

Note: where multiple runs are performed, data are averaged over all systems.

Supplementary Table 4.

Average hydrogen bond data for systems containing gallic acid (GA) solvated with H_2O.[a]

System	Hydrogen bonds (average per GA)			
	GA_{OH} with H_2O	GA_O with H_2O	GA_{OH} with GA	GA_O with GA
GA	1.80 ± 1.08	0.20 ± 0.41	-	-
80BDT$_1$:135GA	1.38 ± 1.05	0.16 ± 0.37	0.19 ± 0.44	0.07 ± 0.26
120BDT$_1$:100GA	1.39 ± 1.04	0.17 ± 0.38	0.19 ± 0.44	0.06 ± 0.24
180BDT$_1$:75GA	1.49 ± 1.06	0.18 ± 0.39	0.11 ± 0.36	0.04 ± 0.20

[a] Based on geometric criteria for the presence of a hydrogen bond with a distance cutoff of 3.0 Å and an angle cutoff of 20°.

Note: where multiple runs are performed, data are averaged over all systems.

Supplementary References

1. Gao, S. et al. Improving the acidity stability of zeolitic imidazolate frameworks by biofunctional molecules. *Chem* **5**, 1597 (2019).
