# Supplementary material for: Particle engineering enabled by polyphenol-mediated supramolecular networks
Source: Nat Commun. 2020 Sep 23;11:4804. doi: 10.1038/s41467-020-18589-0 (PMC7511334; doi:10.1038/s41467-020-18589-0)
Supplement: Supplementary file 2 — Description of Additional Supplementary Files [file 41467_2020_18589_MOESM2_ESM.pdf]

Description of Additional Supplementary Files

File Name: Supplementary Movie 1
Description: **Assembly process of pBDT and polyphenols in water.** Evolution of the complex formation between pBDT (blue) and CAT (red) molecules in water during ~10 ns of MD simulation.

File Name: Supplementary Movie 2
Description: **Disassembly process of pBDT and polyphenols in DMF.** Evolution of the disassembly in DMF of the complex preformed in water between pBDT (blue) and CAT (red) molecules.
